# Host-specific adaptation drove the coevolution of leek yellow stripe virus and *Allium* plants

**DOI:** 10.1128/spectrum.02340-23

**Published:** 2023-09-14

**Authors:** Shusuke Kawakubo, Hangil Kim, Minoru Takeshita, Chikara Masuta

**Affiliations:** 1 Research Faculty of Agriculture, Hokkaido University, Sapporo, Japan; 2 Faculty of Agriculture, Department of Agricultural and Environmental Sciences, University of Miyazaki, Miyazaki, Japan; USDA-San Joaquin Valley Agricultural Sciences Center, Parlier, California, USA

**Keywords:** leek yellow stripe virus, Bayesian phylogeny, host adaptation, virus-host coevolution, RNA silencing suppressor

## Abstract

Host adaptation plays a crucial role in virus evolution and is a consequence of long-term interactions between virus and host in a complex arms race between host RNA silencing and viral RNA silencing suppressor (RSS) as counterdefense. Leek yellow stripe virus (LYSV), a potyvirus causing yield loss of garlic, infects several species of *Allium* plants. The unexpected discovery of an interspecific hybrid of garlic, leek, and great-headed (GH) garlic motivated us to explore the host-adaptive evolution of LYSV. Here, using Bayesian phylogenetic comparative methods and a functional assay of viral RSS activity, we show that the evolutionary context of LYSV has been shaped by the host adaptation of the virus during its coevolution with *Allium* plants. Our phylogenetic analysis revealed that LYSV isolates from leek and their taxonomic relatives (*Allium ampeloprasum* complex; AAC) formed a distinct monophyletic clade separate from garlic isolates and are likely to be uniquely adapted to AAC. Our comparative studies on viral accumulation indicated that LYSV accumulated at a low level in leek, whereas LYSVs were abundant in other *Allium* species such as garlic and its relatives. When RSS activity of the viral P1 and HC-Pro of leek LYSV isolate was analyzed, significant synergism in RSS activity between the two proteins was observed in leek but not in other species, suggesting that viral RSS activity may be important for the viral host-specific adaptation. We thus consider that LYSV may have undergone host-specific evolution at least in leek, which must be driven by speciation of its *Allium* hosts.

**IMPORTANCE:**

Potyviruses are the most abundant plant RNA viruses and are extremely diversified in terms of their wide host range. Due to frequent host switching during their evolution, host-specific adaptation of potyviruses may have been shaped by numerous host factors. However, any critical determinants for viral host range remain largely unknown, possibly because of the repeated gain and loss of virus infectivity of plants. Leek yellow stripe virus (LYSV) is a species of the genus *Potyvirus*, which has a relatively narrow host range, generally limited to hosts in the genus *Allium*. Our investigations on leek and leek relatives (*Allium ampeloprasum* complex), which must have been generated through interspecies hybridization, revealed that LYSV accumulation remained low in leek as a result of viral host adaptation in competition with host resistance such as RNA silencing. This study presents LYSV as an ideal model to study the process of host-adaptive evolution and virus-host coevolution.

## INTRODUCTION

Host adaptation of a virus is a crucial step for virus evolution. Plant viruses generally have a wider host range than animal viruses and often show broader diversity according to how a virus adapts to certain hosts as a specialist or generalist (1). Viral host switching is affected by not only virus intrinsic factors but also extrinsic factors such as environmental or ecological features and diversification of host species (2). Thus, for a virus, adapting to a new host is considered a long-term evolutionary process through a complex history of virus-host interactions (3).

The genus *Potyvirus* (family *Potyviridae*) currently comprises 195 species of plant RNA viruses that can infect a wide range of monocots and eudicots and is thus one of the most diversified viral groups (4, 5). Potyviruses are transmitted mainly by aphids in a nonpersistent manner. The common ancestor of potyviruses was estimated to have diverged approximately 7,250 years ago in Southwest Eurasia or North Africa, with radiation events 6,600 y ago (6, 7); and frequent gain- and loss-of-function mutations then led to a shift in their host ranges (8). They can infect and cause large yield losses for a variety of economically important crops and ornamental plants.

*Allium* species such as garlic (*Allium sativum*), onion (*A. cepa*), and leek (*A. ampeloprasum*) are popular, historical crops cultivated worldwide; they are used not only for food and seasoning but also for medicinal purposes. In addition to garlic, an *Allium* crop producing oversized bulbs termed great-headed (GH) or elephant garlic is widely cultivated as a substitute for garlic. Although GH garlic resembles garlic, it is classified as a variety of *A. ampeloprasum* (9). The origins of *Allium* plants remain unclear although onion and garlic were first domesticated in the mountainous areas of Central Asia. Leek is an ancient vegetable that was consumed by Romans and other ancient cultures (10). It was later introduced to Wales, where it is now the national vegetable. Garlic was originated in West to Central Asia and cultivated in the Mediterranean and other areas more than 5,000 years ago (11), while leek and garlic probably emerged in similar geographic areas (10). GH garlic is also considered originated in the Mediterranean region even though its species concept is still under debate (12).

Leek yellow stripe virus (LYSV), one of the most devastating potyviruses infecting garlic, causes a severe dwarf disease, which reduces bulb mass by up to 88% (13). LYSV infects not only garlic but also leek and onion (14 – 16). Unlike leek and onion, cultivated garlic does not usually produce true seeds, so plants are propagated vegetatively and any clones from LYSV-infected stock will also be infected. In production fields, garlic plants are often infected with several viruses including LYSV, onion yellow dwarf potyvirus (OYDV), allexiviruses (garlic viruses A, B, C, D, E, and X), and carlaviruses. LYSV was first isolated from leek (*A. ampeloprasum*) in the Netherlands in 1978 (15). Yellow stripe diseases of leek have been frequently reported in Germany since 1937; the causal agent was later identified as LYSV (17). LYSV infection of commercial garlic plants was first officially documented in 1987 (18). Until LYSV was discovered in garlic, the plant symptoms induced by LYSV were thought to be caused by strains of OYDV (15).

LYSV isolates can be divided into two types: N-type and S-type based on the presence of a 68-amino-acid (AA) deletion in the N-terminal half of the P1 protein (P1) (14, 19, 20). Although the function of P1 has not been completely elucidated, P1 has been found to function as a cofactor for the potyvirus RNA silencing suppressor (RSS), helper component proteinase (HC-Pro) (21). In our recent study, we demonstrated that P1 alone can acquire RSS activity, especially when the N-terminal region is deleted (20), and that P1 greatly contributes to the viral adaptation to its host, thus serving as one of its host determinants.

In the present study, from a local market in Japan, we obtained leek and GH garlic plants that had been infected with LYSV. We also found an LYSV-infected garlic-like *Allium* plant at the market; its bulb size was intermediate to those of the garlic and GH garlic, although it was being sold as regular garlic. Later, we genetically identified this plant as an interspecific hybrid of leek, GH garlic, and garlic. Bayesian phylogenetics and phylogenetic comparative methods have been shown as powerful approaches to elucidate the evolutionary backgrounds of many plant pathogens (22 – 24). We here demonstrated that LYSV isolates infecting *Allium* plants showed clear phylogenetic relationships between their evolution and *Allium* plant speciation, which have been rarely reported for potyviruses (8). We discussed how the evolutionary context of LYSV has been shaped by its adaptation to *Allium* host plants during their coevolution, and then proposed that the LYSV evolution in *Allium* plants could have been greatly affected by the RSS activity of the virus.

## RESULTS

### The garlic-like plant from a market may be an interspecific hybrid of several *Allium* species

**Fig 1:**
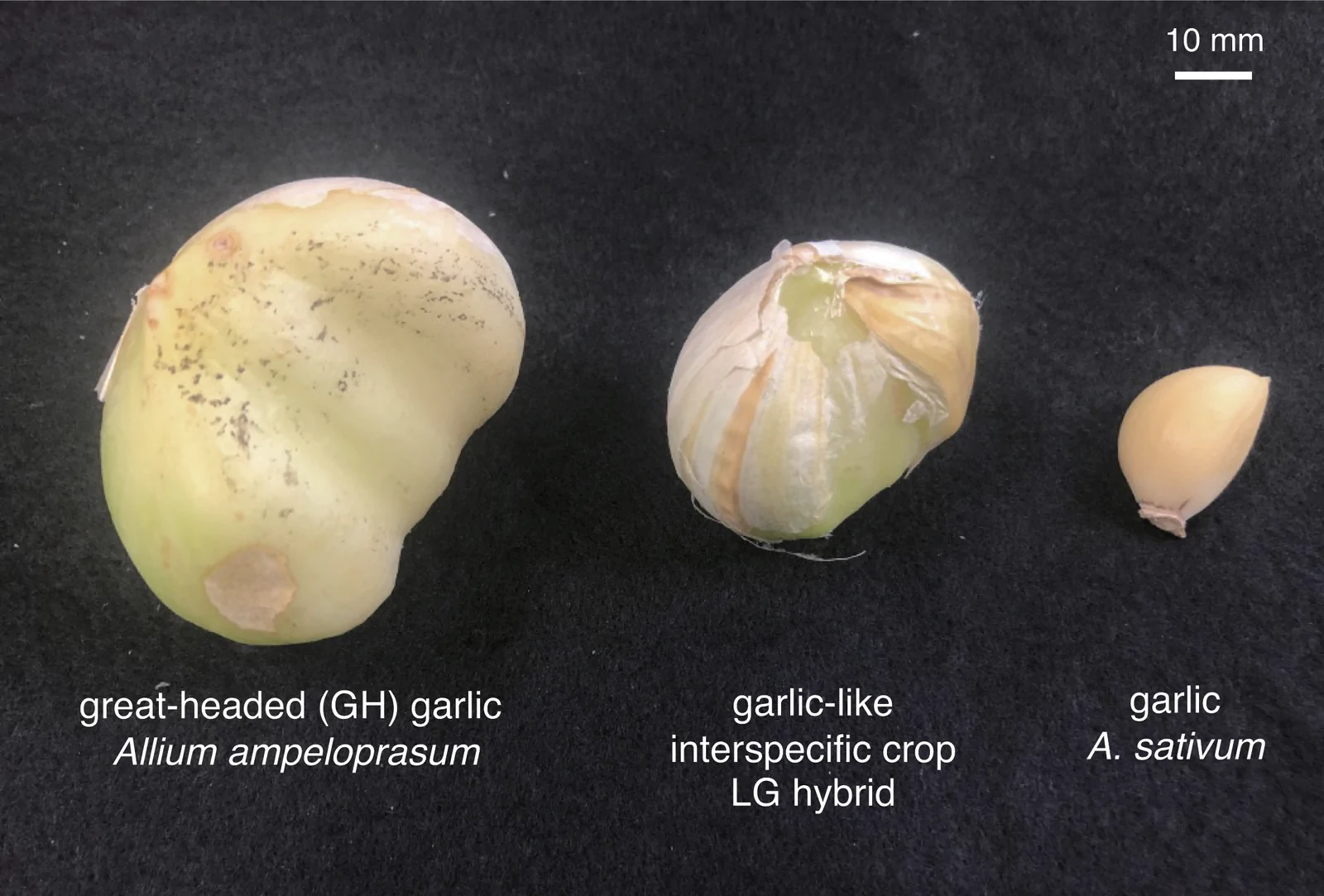
Bulbs of *Allium* plants used in this study.

Although labeled by the producer and sold as “garlic,” this garlic-like plant had a bulb that was intermediate in size between those of GH garlic and typical garlic (Fig. 1). We thus suspected it might be another *Allium* species and explored its genetic background. Because the amino acid sequences encoded by the alliinase genes in *Allium* species contain species-specific amino acid residues (25), we identified the alliinase genes in this plant using our RNA-Seq data. Unlike our expectation, two amino acid residues (Fig. S1, positions 38 and 58) that can distinguish leek and garlic were heterozygously detected in the alliinase gene of the garlic-like plant (Fig. S1). In addition, two other amino acid residues (Fig. S1. positions 52 and 55) that can distinguish GH garlic and garlic were also detected at the same rate. We also examined the sequences of the nuclear ribosomal internal transcribed spacer (ITS) region of this plant and confirmed the hybrid nature of this plant among garlic, leek, and GH garlic (Table S1). These results thus suggest that this garlic-like plant may be an interspecific hybrid of garlic, leek, and GH garlic; hereafter, we refer to this garlic-like plant as an “LG hybrid.”

### LYSV detection from the LG hybrid, GH garlic, and leek and comparison of viral levels

**Fig 2:**
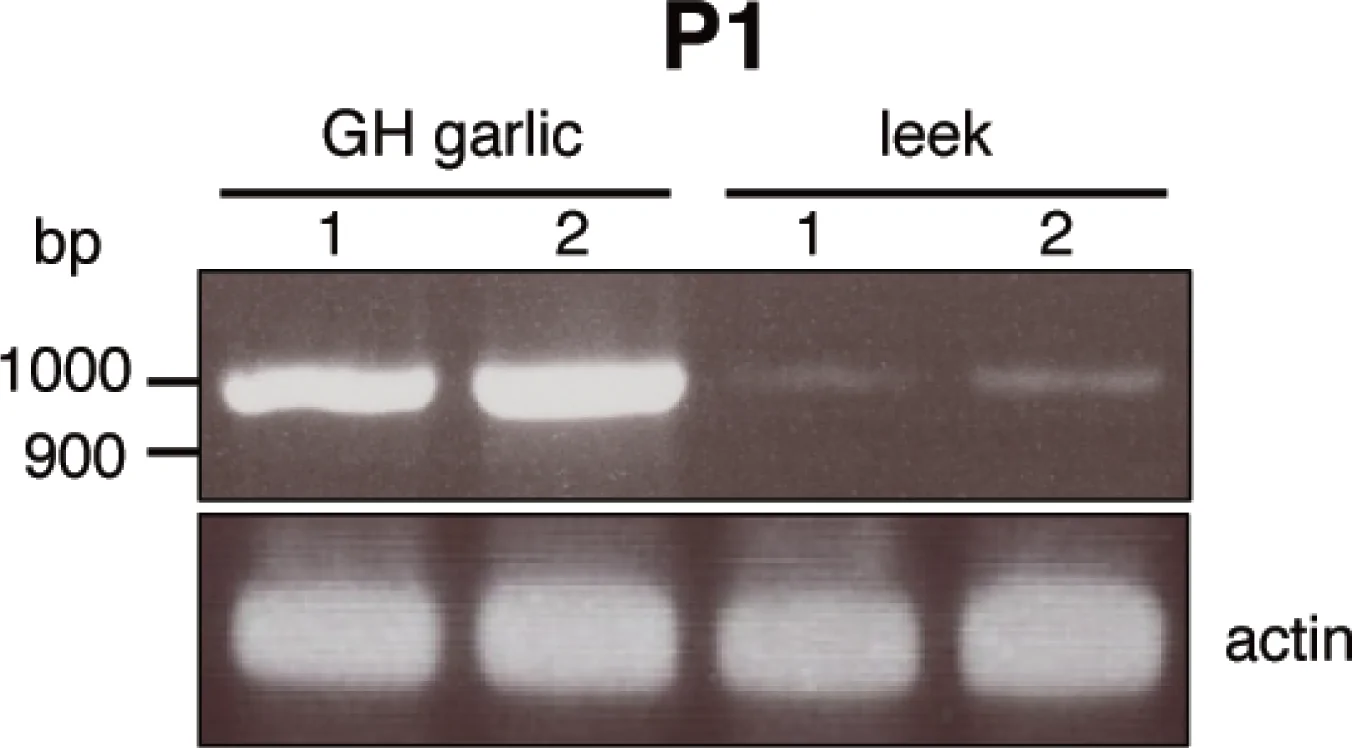
RT-PCR to detect LYSV from GH garlic and leek. The PCR products were analyzed after 40 cycles. The actin gene was used as an internal control. The lane number represents each individual plant. Primers are listed in Table S3.

We then cloned the LYSV P1 gene from the LG hybrid and determined the nucleotide sequence because the P1, which serves as a key element of viral host adaptation, was our initial interest. As the result of a BLASTn search, we found that the sequence best matched that of an Indonesian LYSV isolate from leek (MW854277), but with only 84% shared identity. We then performed pairwise nucleotide comparisons of the sequences of garlic LYSV isolates in Japan using EMBOSS-NEEDLE (26). However, the P1 sequence shared only 51% identity with that of garlic isolates. We then obtained the entire nucleotide sequence of the infected LYSV from our RNA-Seq data. To confirm the assembled LYSV sequence from RNA-Seq analysis, we PCR-amplified the viral genome based on the high-throughput sequencing (HTS) information and redetermined the nucleotide sequence. We next tried to PCR-amplify some LYSV genes from GH garlic and leek plants purchased at the market. The LYSV genes (P1, HC-Pro, and coat protein [CP]) from GH garlic were readily amplified using a simple one-step RT-PCR (30–35 cycles), but the same RT-PCR did not generate any specific PCR product from leek and only amplified a very low level of the P1 after 40 cycles (Fig. 2). These results suggest that LYSVs are certainly present in leek, but at very low levels. Our RT-PCR and subsequent nested-PCR to amplify the CP gene confirmed that LYSV was present at higher levels in GH garlic and LG hybrid than in leek (Fig. S2).

### LYSV population was characterized by their host plants rather than geographic area

**Fig 3:**
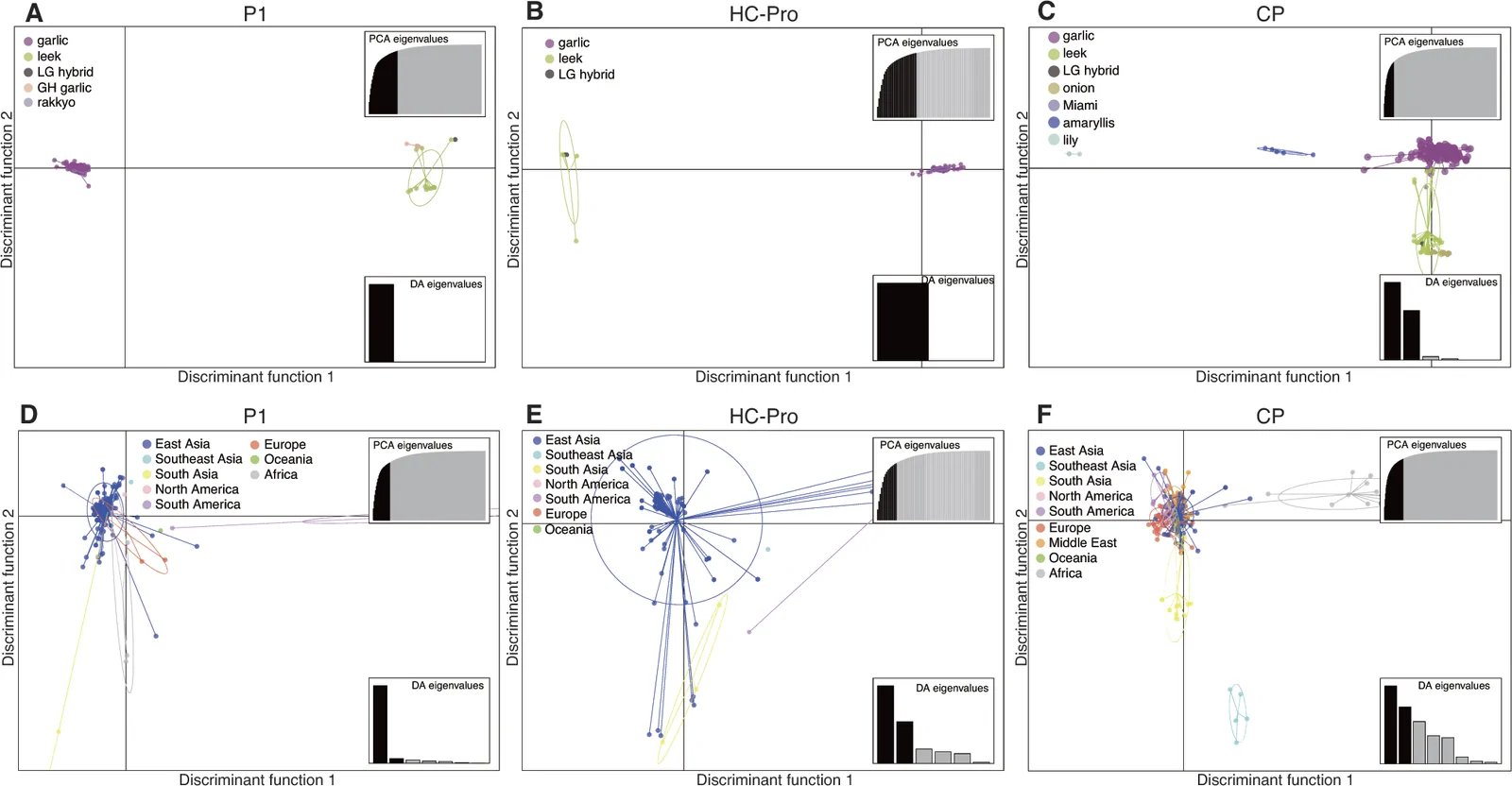
Scatter plots of results of discriminant analysis of principal components using the viral nucleotide sequences of P1 (**A and D**), HC-Pro (**B and E**), and CP (**C and F**). Discriminant analysis of LYSV isolates with (**A–C**) host plants or (**D–F**) geographic areas used as a population trait. The circles indicate 95% inertia ellipses. Rakkyo is a common name for *Allium chinen*se G. Don; Miami is an ornamental onion of *Allium* species.

We next explored the genetic composition of the LYSV population using all the available LYSV sequences in GenBank. We first applied a discriminant analysis of principal components (DAPC) to assess how much of the genetic variation of the LYSV population is explained by the host plant or geographic area. As we described, LYSV P1 has been demonstrated to work as an enhancer of the RSS activity of HC-Pro (20). The interaction between HC-Pro and CP is necessary for aphid transmission of the virus (27, 28). Considering the interactions among the three proteins, we used these three genes (i.e., P1, HC-Pro, and CP) for our DAPC analysis. When the host species were used as a population trait, the DAPC analyses for all the P1, HC-Pro, and CP genes clearly showed genetic separation of garlic and leek based on the first or second discriminant function (Fig. 3A through C). However, when the LYSV isolates were analyzed based on the sampled geographic areas, they are hardly separated (Fig. 3D through F). These results thus demonstrate that leek LYSVs may have adapted to their host plants rather than to the geographic areas of the plants; the leek isolates used in this analysis were collected in Indonesia, Japan, Netherlands, Serbia, Turkey, and Vietnam.

### Comparison of the deletion sequences in the P1 protein and tip-dating time-scale analysis

**Fig 4:**
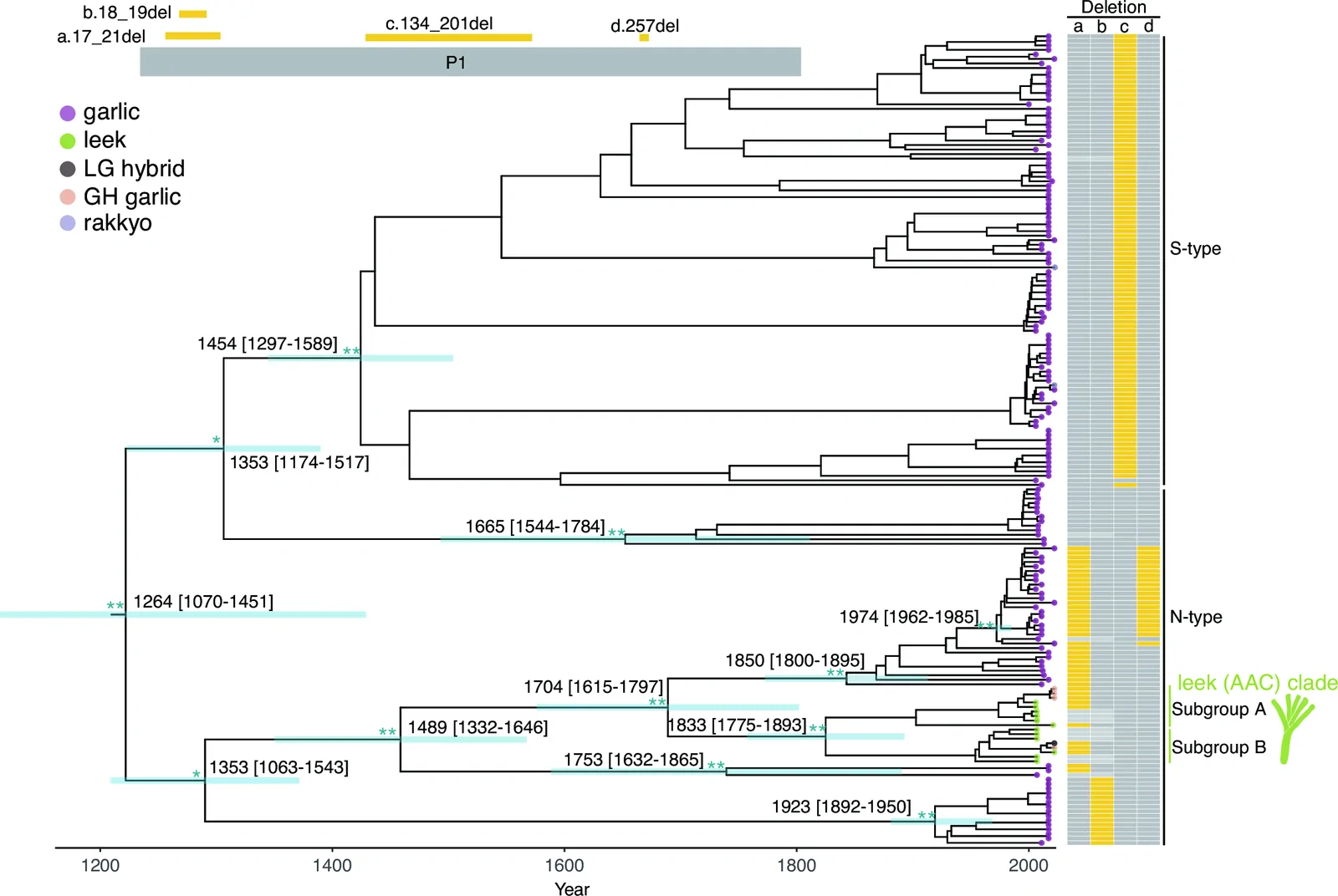
Time-scaled maximum clade credibility tree of the P1 gene. Colors at the tipping points represent the hosts shown in the color key. The *x*-axis is the timescale in years. The blue bar at each node of interest is the 95% Bayesian credible interval for the inferred time. Sequence deletions in each isolate are shown in the diagram at the top as an array of the vertical box on the right (yellow, presence of deletion; gray, absence of deletion; light gray, missing values due to partially available sequences). * posterior probability >0.7; ** posterior probability >0.9.

We constructed a time-calibrated Bayesian phylogenetic tree with a plot of the various deletions in the P1 protein (Fig. 4). Our analyses confirmed the presence of temporal structure in the data sets based on a date-randomization test using clustered permutation (Fig. S3). All the leek LYSV isolates clustered in a distinct clade from garlic LYSV. The LYSV isolated from the LG hybrid was also in this leek clade. All the leek isolates lacked the 68-AA deletion (134_201del) and were N-type isolates. The most recent common ancestor (MRCA) for the leek clade was predicted to be dated back to 1833 (95% credible interval [CI]: 1775–1893). In addition, the leek clade was further divided into two highly diverged subgroups with a high posterior probability (PP) (Fig. 4). Our LYSV isolates from GH garlic also clustered in the leek clade; three sequences belonged to subgroup A and one to subgroup B. Most of the N-type isolates had a 4-AA deletion (17_21del), whereas a minor sister clade had a 2-AA deletion (18_19del) at the same position as that of the 4-AA deletion. The branching of this sister clade was supported with a relatively low PP. One monophyletic clade that contained another deletion of 1-AA (257del) with the 4-AA (17_21del) deletion was inferred to have diverged in 1974 (95% CI: 1962–1985) within the N-type population. All the S-type isolates had just one deletion site (68-AA deletion, 134_201del), while one monophyletic clade that did not have the 134_201del deletion was a direct sister lineage to the S-type. The MRCA of all LYSV isolates was inferred to have diversified in 1264 (95% CI: 1070–1451).

### Repeated motif in the P1 protein

**Fig 5:**
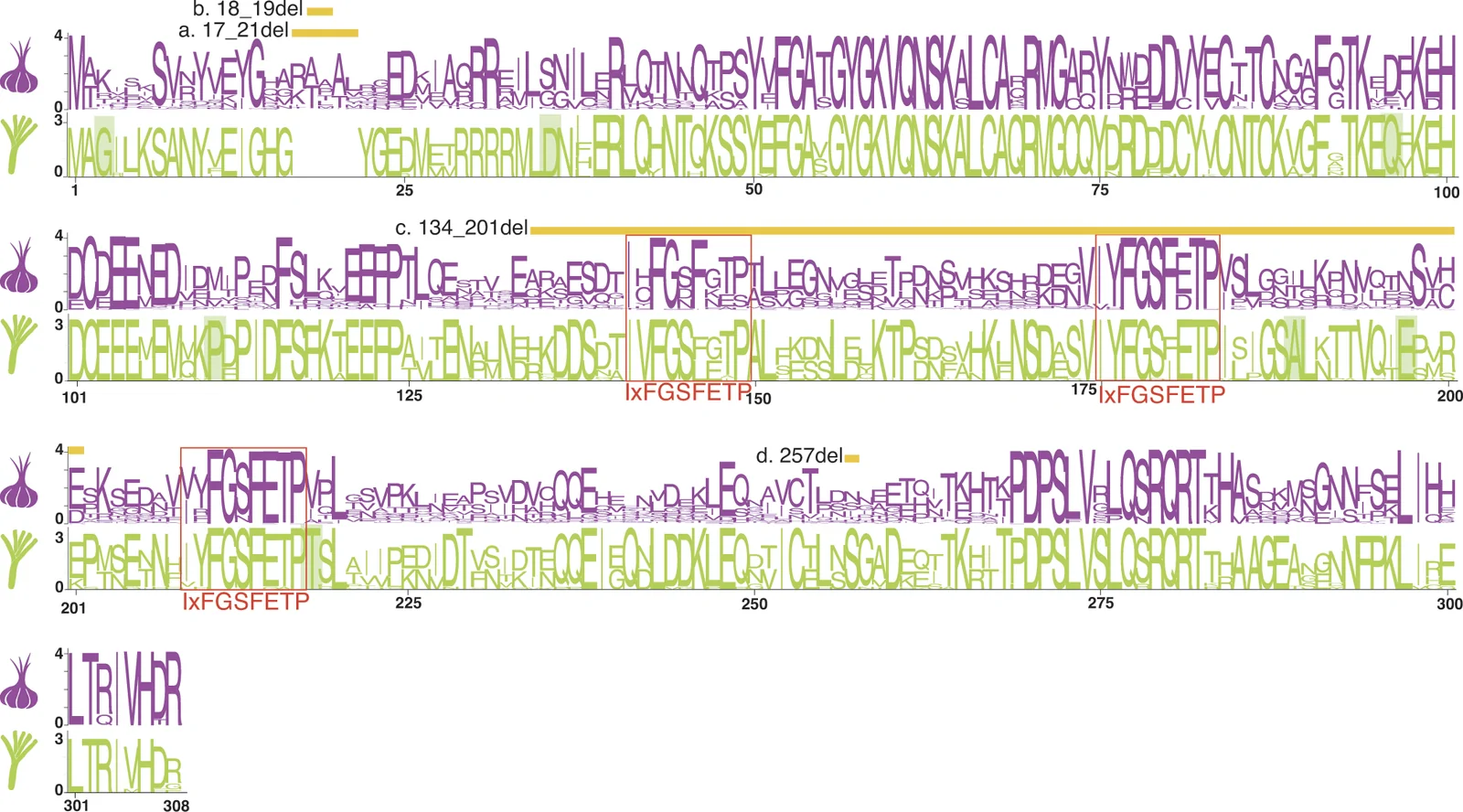
Amino acid sequences of the N-terminal half of the P1 proteins. The sequences shown in purple on the top indicate LYSV isolates from garlic, and in green at the bottom from leek. The *x*-axis indicates translated amino acid sequence positions for the LYSV genome (AB194649). The *y*-axis indicates the bit score for cross-entropy at the sequence position. The conserved repetitive motifs are boxed in red. The yellow bars (a–d) correspond to the deletion positions shown in Fig. 4. Plots were created using WebLogo (https://weblogo.berkeley.edu).

Considering the high sequence variabilities in the P1 protein due to the multiple deletions and the P1 phylogeny suggesting host adaptation, we searched for unique motif(s) in the P1 protein that might be associated with host specificity. We found one previously reported conserved potyviral motif (IxFG) in the P1 protein (29) in both garlic and leek LYSVs, but as a longer form (IxFGSFETP). As shown in Fig. 5; Fig. S4, this conserved motif appeared three times in the N-type isolates. Because this motif corresponds to the 68-AA deletion (134_201del), the S-type LYSVs with this deletion had only one unit of this motif. There were 7 AA positions uniquely conserved to the leek LYSV isolates (Fig. 5).

### Ancestral state reconstruction in a Bayesian phylogenetic framework

**Fig 6:**
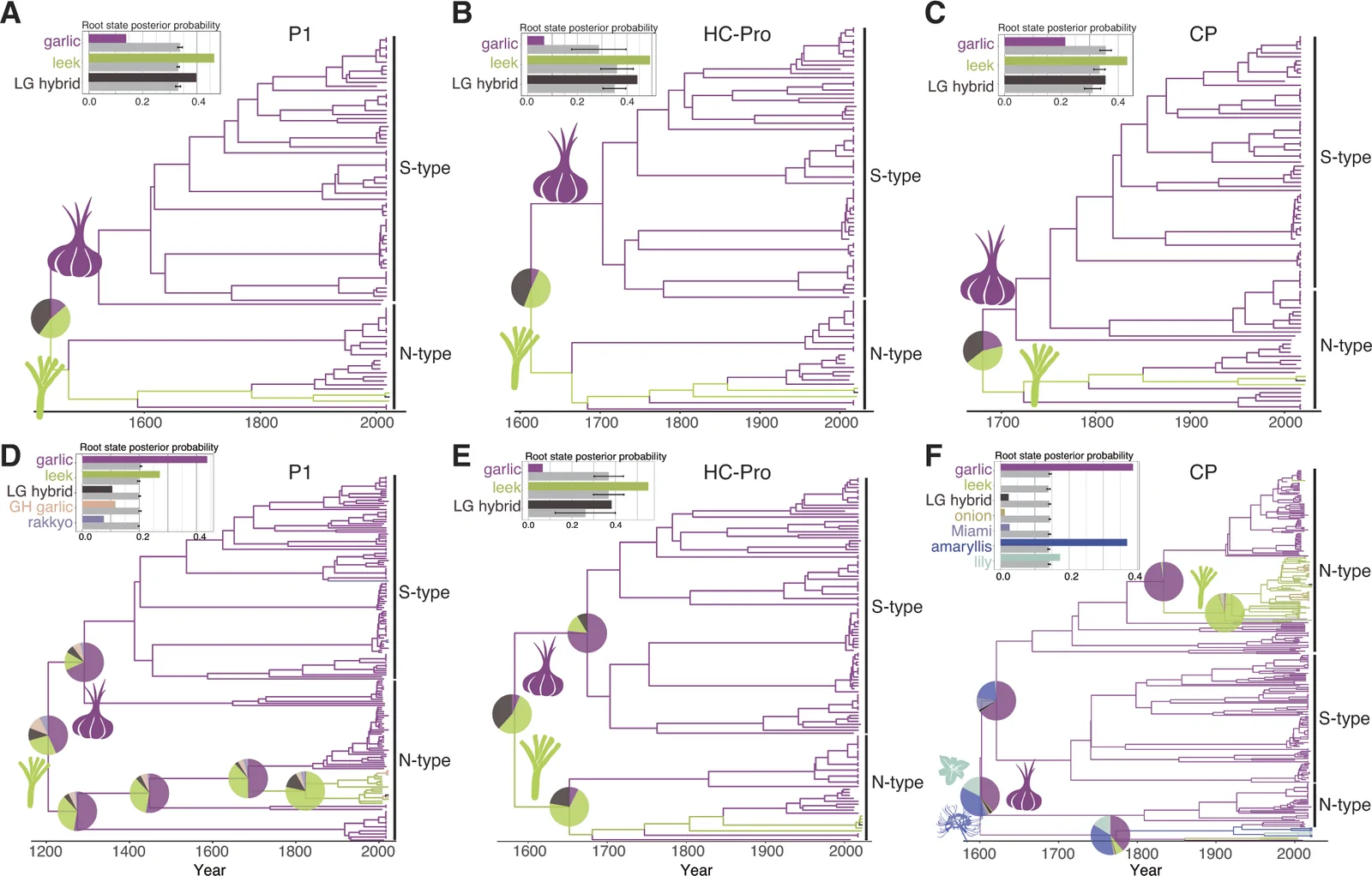
Time-scaled maximum clade credibility trees for the P1 (**A and D**), HC-Pro (**B and E**), and CP (**C and F**) data set. (**A–C**) is based on the filtered data set that shared the same taxon set (*n* = 93) and (**D–F**) is based on the total available isolates in each gene (P1, *n* = 179; HC-Pro, *n* = 123; CP, *n* = 300). Branch colors indicate the inferred results of the ancestral state reconstruction of host plants. Pie charts indicate the posterior probabilities of the host plants inferred as the ancestral state. The *x*-axis is scaled in years. The histogram (top left) shows the posterior probability of the root for each host plant; the gray bars indicate the posterior probability with error bars obtained from randomized replicates of the tip state.

Because our phylogenetic tree for the P1 gene showed a unique group of leek isolates as a sister clade to the major clade of N-type isolates, we were curious how the hosts of LYSV had changed during viral evolution. To examine the transition of the ancestral hosts of LYSVs, we performed an ancestral state reconstruction in a Bayesian phylogenetic framework for the P1, HC-Pro, and CP genes (Fig. 6). To minimize the misinterpretation due to bias in the number of sequences for each gene, we first filtered out sequences so that they share the same taxon (*n* = 93) in the data set. As a result, the leek was inferred to be the most probable ancestral host for LYSVs based on all three genes with the highest PPs of 0.46 for P1, 0.49 for HC-Pro, and 0.43 for CP (Fig. 6A through C). On the other hand, the PPs of garlic at the root were very low: 0.14 for P1, 0.07 for HC-Pro, and 0.21 for CP. The MRCA(s) of these taxon sets were inferred to have been diversified in 1439 (95% CI: 1223–1634) for P1, in 1614 (95% CI: 1477–1732) for HC-Pro, and in 1680 (95% CI: 1562–1785) for CP (Fig. 6).

When we performed the same analysis using all the available isolates, the data set contained more than twice the number of sequences than in the previous analysis (P1, *n* = 179; HC-Pro, *n* = 123; CP, *n* = 300) (Fig. 6D through F). For HC-Pro, the most probable ancestral host was consistently inferred to be leek with PP of 0.55 (Fig. 6E) while they were low for P1 and CP (0.27 and 0.01, respectively) (Fig. 6D through F). When the P1 sequence was used, the ancestral host at the root was inferred as garlic with a PP of 0.44. For the CP tree, although garlic had the highest PP at the root with 0.39, amaryllis, which is not *Allium* species, was inferred as a possible ancestral host with a PP of 0.37. The CP data set with LYSV isolates of lily showed the host state PP of 0.17 at the root. However, the tree topology and rooting position for CP were totally different from those of the other genes and the filtered data set of the CP gene (Fig. 6F). These discrepancies may be due to different numbers of the sequences used; among the total of 300 isolates included in the CP data set, 141 isolates were not included in the data set for the other genes. The phylogenetic trees with the tip label correspondence are summarized in Table S2 and Fig. S5 through S7 for reference.

### Diversification rate shifts and host-specific adaptation in the LYSV evolution

**Fig 7:**
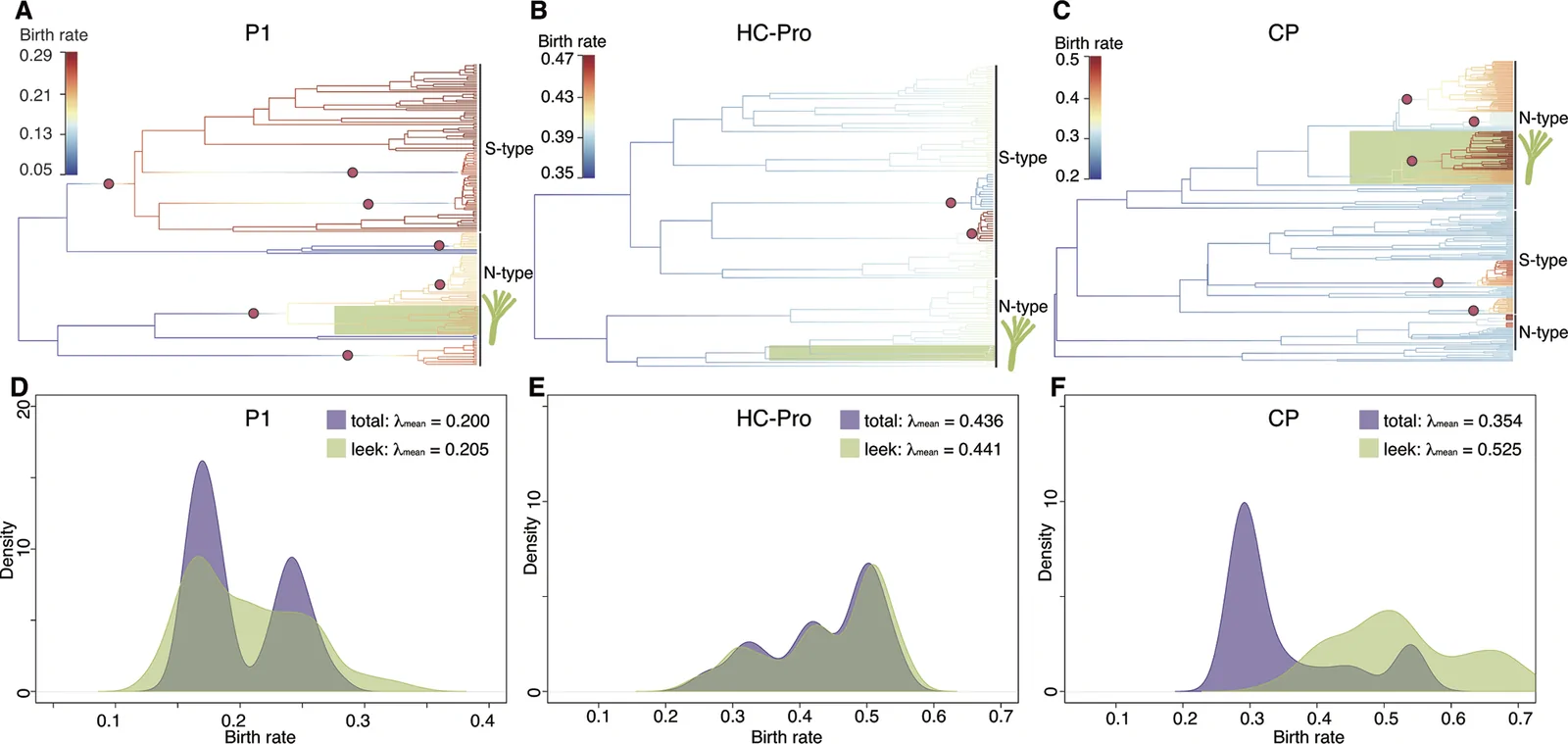
Diversification dynamics during LYSV evolution inferred for the P1 (A and D), HC-Pro (B and E), and CP (C and F) genes. (A–C): Mean phylorate plots whose tree topologies were equivalent and derived from the BEAST output as shown in Fig. 6D through F. The leek (AAC) clade is shaded in green. Red circles on tree branches indicate epochs of rate shifts for each distinct shift configuration inferred from the maximum a posteriori probability. (D–F): Kernel density of birth rate values sampled through the Markov chain Monte Carlo procedure. Means are given in the keys.

Based on the time-calibrated trees and the inferred ancestral host transition, we were motivated to examine whether the diversification rate had changed during the LYSV evolution. The maximum clade credibility (MCC) trees in the Bayesian phylogenetic analysis described above were transformed to an ultrametric form and subsequently reanalyzed using the BAMM programs (30, 31). The results indicated that the diversification rate had indeed shifted multiple times during LYSV evolution (Fig. 7). The emergence of the S-type LYSVs in the P1 gene analysis showed a clear shift with higher birth rates, and the leek clade in N-type had also branched with the distinct birth rates (Fig. 7A). In addition, while the HC-Pro tree did not show any major difference in birth rate between leek and garlic (Fig. 7B), the CP tree showed that LYSVs in the leek clade had apparently higher birth rates than those of the others (Fig. 7C). These results indicate that viral fitness and subsequent diversification of LYSVs changed multiple times during the virus evolution.

### Viral RSS activity differs depending on the host plant species

**Fig 8:**
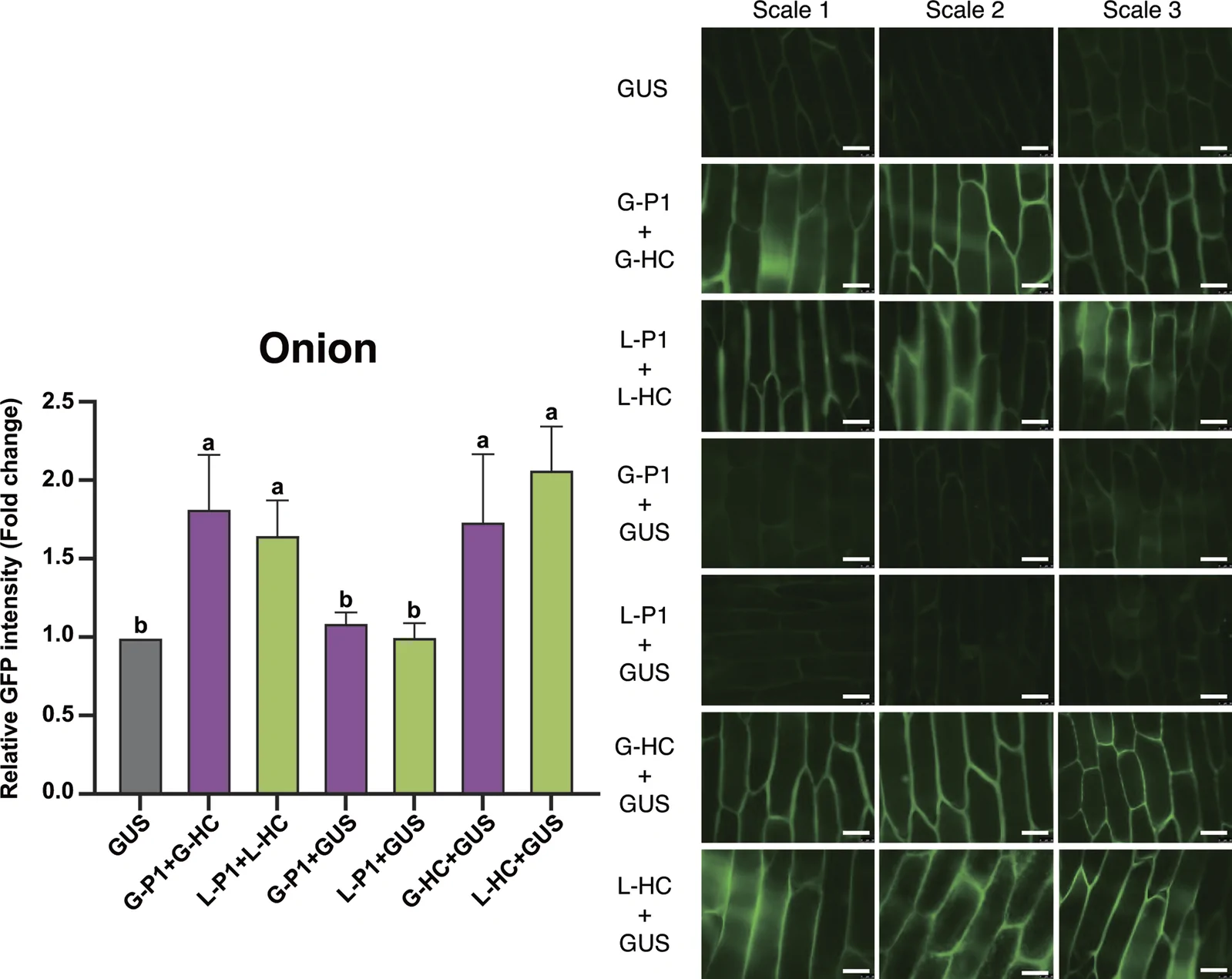
RSS activity of LYSV P1 and HC-Pro at 3 days post-agroinfiltration (dpa) using onion epidermis. P1 and HC-Pro of garlic strains (**G-P1 and G-HC**) and leek strains (**L-P1 and L-HC**) were co-expressed with the *GFP* gene. Greater GFP fluorescence intensity indicates greater RSS activity. Three different onion scales (scales 1–3) are shown. Relative mean fold changes in GFP fluorescence intensity, calculated using the LAS AF software (Leica), are shown in the barplot with the value of *GUS* set to 1.0. Fill colors in the barplot represent the LYSV strains (purple, garlic strain; green, leek strain). Fold change values were log-transformed, then analyzed by one-way ANOVA (*P* < 0.0001) followed by Tukey’s multiple comparison test. Different letters above the bars indicate a significant difference between means (*P* < 0.05).

**Fig 9:**
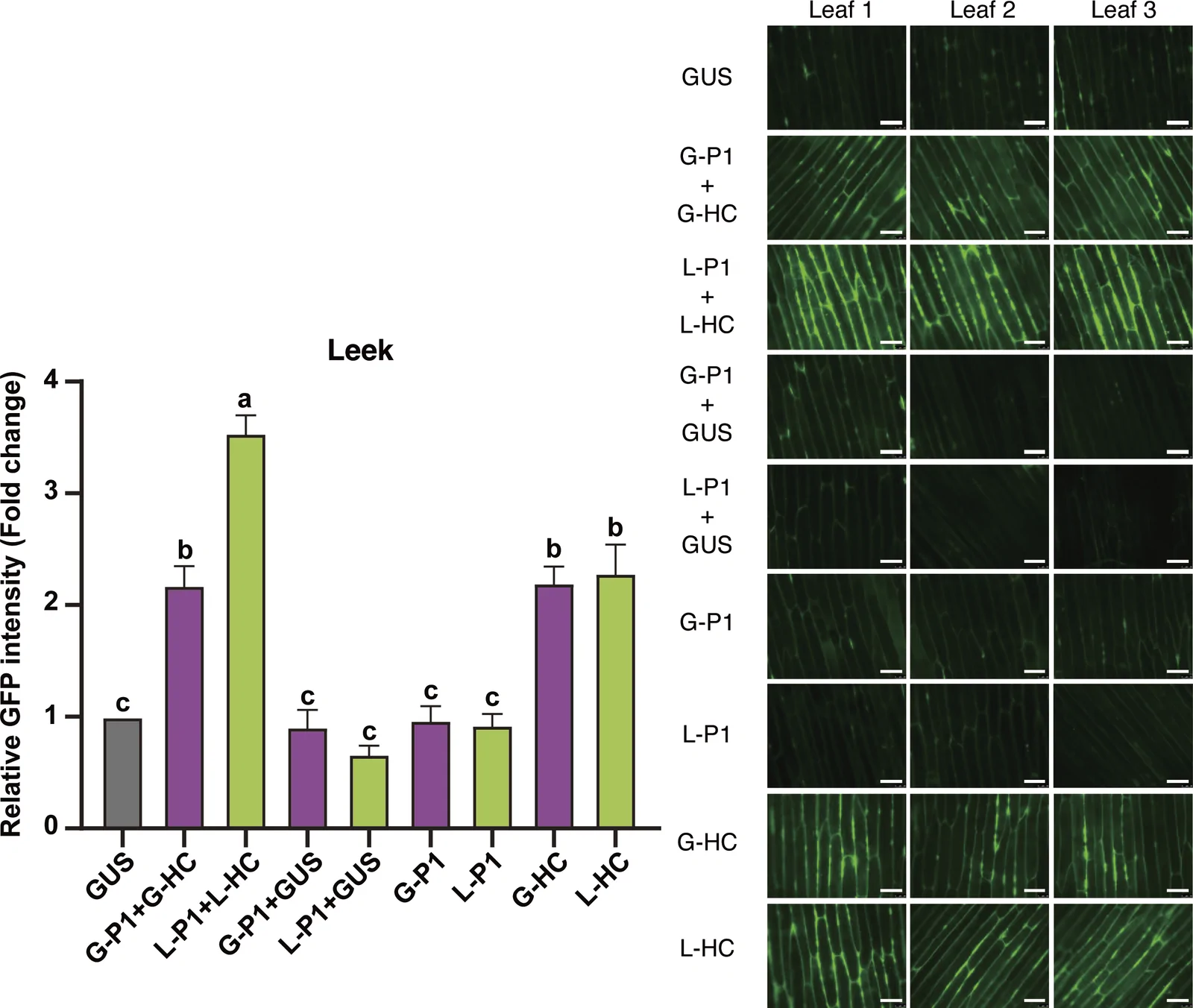
RSS activity of LYSV P1 and HC-Pro at 3 days post-agroinfiltration (dpa) using leek leaves. P1 and HC-Pro of garlic strain (**G-P1 and G-HC**) and leek strain (**L-P1 and L-HC**) were co-expressed with the *GFP* gene. Greater GFP fluorescence intensity indicates greater RSS activity. Three different infiltrated leaves (leaves 1–3) are shown. Relative mean fold changes in GFP fluorescence intensity, calculated using the LAS AF software (Leica), are shown in the barplot with the value of *GUS* set to 1.0. Fill colors in the barplot represent the LYSV strains (purple, garlic strain; green, leek strain). Fold change values were log-transformed, then analyzed by one-way ANOVA (*P* < 0.0001) followed by Tukey’s multiple comparison test. Different letters above the bars indicate a significant difference between means (*P* < 0.05).

P1 protein was previously reported to enhance the RSS activity of HC-Pro (21). Because the LYSV P1 protein was recently found to also have RSS activity (20), we first investigated whether P1 of leek LYSV and garlic LYSV actually have RSS activity in their natural host of two *Allium* species, onion and leek. In this experiment, we used the transient expression system using the agroinfiltration method. Unfortunately, we found that agroinfiltration into garlic tissues was not easy because garlic leaf tissue was too rigid to be infiltrated. In both onion epidermis and leek leaves, P1 of leek LYSV and garlic LYSV did not show any distinct RSS activity (Fig. 8 and 9). We also observed similar results using *Nicotiana benthamiana*, which is the most commonly used assay plant (Fig. S8). We then examined the RSS activity of HC-Pro by expressing it alone or co-expressing with P1. When onion epidermises were used, HC-Pros of both garlic LYSV and leek LYSV showed distinct RSS activity, but the co-expression of P1 did not show any significant enhancement of RSS activity of HC-Pro (Fig. 8). However, when leek leaves were used, the RSS activity of leek HC-Pro was clearly enhanced by the co-expression with P1, although the original RSS activity of HC-Pro itself was similar between leek LYSV and garlic LYSV (Fig. 9). P1 of garlic LYSV did not have enhancer activity for HC-Pro in leek and onion (Fig. 8 and 9). When *N. benthamiana* was used*,* garlic HC-Pro itself showed significantly higher RSS activity than that of leek HC-Pro, and the co-expression of leek P1 and leek HC-Pro showed significantly lower RSS activity than the co-expression of garlic P1 and garlic HC-Pro (Fig. S8). Taken together, these results suggest that the RSS activity of LYSV HC-Pro differs depending on the assay plant species and that P1 and HC-Pro from leek LYSV may have evolved to function cooperatively in leek tissues.

## DISCUSSION

Our discovery of a garlic-like plant, which we later determined as a hybrid among garlic, leek, and GH garlic, motivated us to initiate this study (Fig. 1; Fig. S1; Table S1). Because LYSV levels were high in this hybrid plant, we easily determined the entire nucleotide sequence of LYSV by RNA-Seq. The obtained LYSV sequence was quite different from those previously reported for garlic LYSVs, but rather similar to that of LYSV infecting leek. We then tried to isolate an LYSV from leek and GH garlic and found that LYSV accumulated at a very low level in leek, whereas it is quite abundant in GH garlic (Fig. 2; Fig. S2). So what is the reason for the difference in LYSV accumulation between leek and GH garlic (or garlic)?

In the several studies on the phylogenetic relationships among *Allium* species based on their transcriptomes, or nuclear and chloroplast sequences (32 – 35), garlic and leek shared the same common ancestor; therefore, garlic is more closely related to leek than the other *Allium* species including onion (*A. cepa*) and *A. fistulosum*. GH garlic and leek are taxonomically categorized as species in the *A. ampeloprasum* complex (AAC) with kurrat and pearl onion (9, 36, 37). The plants in the AAC are cytogenetically diverse in terms of chromosomal polyploidy; leek is tetraploid (2*n* = 4 × =32) while GH garlic is hexaploid (2*n* = 6 × =48) (36). Ariga et al. showed that the amino acid sequences of the enzyme alliinase were unique in each *Allium* species. Interestingly, GH garlic has two unique amino acids for garlic that are not conserved in leek (25). More importantly, the LG hybrid, which we purchased, heterozygously shared unique amino acids with both leek and GH garlic (Fig. S1), suggesting that this hybrid plant has unique amino acids for garlic derived from GH garlic. Further comparison based on the ITS regions among *Allium* plants also supported that this plant had been generated through cross-hybridization (Table S1).

**Fig 10:**
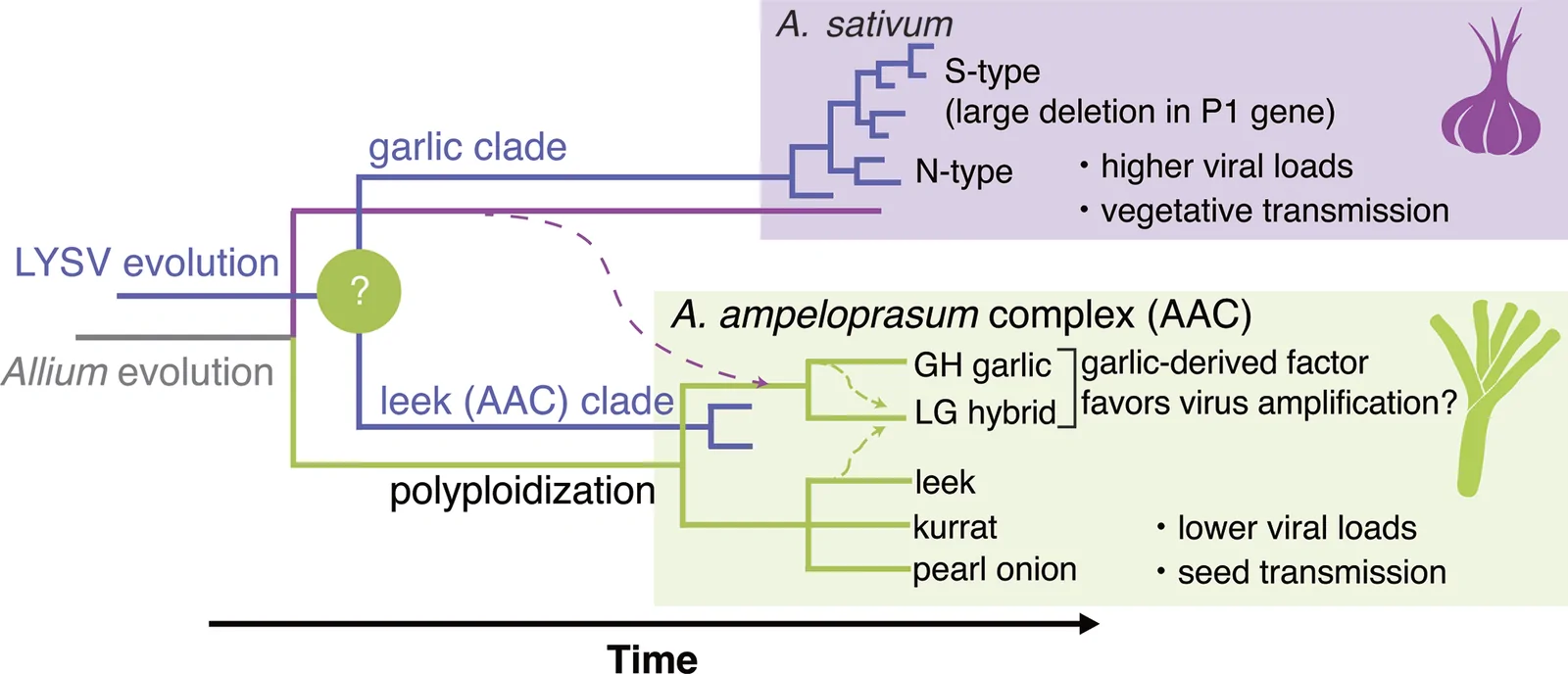
Possible scenario of the coevolution of LYSV and *Allium* species plants. The dashed line indicates a potential horizontal chromosome transfer event among *Allium* species.

The reason why leek LYSV maintains a low level of accumulation in leek tissues is not clear, but may be a result of adaptive evolution acquired after leek LYSV had diverged from the ancestral virus. It may be that there are no host factors in leek that allow LYSV to replicate efficiently. Alternatively, there may be some resistance mechanism(s) functioning in leek that prevents LYSV from accumulating at high levels. Thus, leek LYSV may be characterized as a latent virus, which does not produce disease symptoms in infected plants. Latent plant viruses will not disappear in infected plants even at low levels, which will not be a demerit for their survival (38). Therefore, we consider that some garlic-derived factor(s) might somewhat promote LYSV multiplication/accumulation to increase the viral titer (Fig. 10).

Our phylogenetic analyses revealed that leek LYSVs formed a distinct clade together with those from GH garlic and the LG hybrid (Fig. 4 and 6). In this context, the leek clade may be referred to as the AAC clade. A few previous studies on LYSV phylogenetics also described a distinct clade for leek LYSVs (14, 39). We here further demonstrated that this clade is not only for leek but also for the AAC. In our phylogenetic analysis of the CP data set, onion LYSVs from Turkey were also in the same AAC clade (Fig. 6F). Considering that pearl onions have been commonly cultivated in Europe, it may be worth exploring whether the Turkish onion infected with LYSV is related to one of the AAC species.

*Allium* crops were suggested to have been introduced to the Mediterranean area from Central Asia by Marco Polo (40) in the 13th century; thus, the area would be a secondary center for the spread of *Allium* species. Such human interventions can greatly affect the evolution of viruses, as exemplified by many plant viruses (23, 41). Based on our tip-dated molecular clock analysis, we inferred that the first divergence of LYSV was in 1264 (95% CI: 1070–1451) based on the P1 sequences (Fig. 4). The evolutionary timescale of LYSV seems to coincide with the diversification of garlic and leek (Fig. 10). However, the divergence time based on HC-Pro and CP or the filtered data set of P1 was dated as fairly recent (Fig. 6). We were aware that some discrepancy among the inferred timescales had been sometimes reported for potyviruses when analyzing different genes or different sizes of the data set for the same gene (23, 41). In our phylogenetic analysis of the three different genes on the same viral genome (i.e., the filtered data set), we inferred that LYSV might have been diversified from the common ancestor that was adapted to leek (Fig. 6A through C). This inference does not seem to be robust because the analysis of the data set including all available sequences for P1 and CP (Fig. 6D and F) showed garlic as an ancestral host with higher PP than leek. To further explore the robustness of the phylogenetic tree, we also created phylogenetic trees for the potyviral NIb gene (Fig. S9 and S10), considering that RNA-dependent RNA polymerase (NIb) phylogeny is often used to provide robust results because conserved sequences are often found among related viruses (42). As a result, the ancestral host of LYSV based on the NIb gene was inferred as garlic (Fig. S9 and S10). Phylogenetic incongruence among the analyzed genes is commonly known as the difficulties especially when several genes in the same species are under different selection pressures (43). In our study, all the phylogenetic trees indicated that interspecies virus transmission between garlic and AAC rarely occurred. This host selectivity seems to have established a long-term, independent circulation of LYSV only within garlic or AAC populations, further affecting viral host adaptivity. Even though the ancestry of LYSV has been not perfectly demonstrated, at least HC-Pro appears to have evolved from the ancestral LYSV that was adapted to leek. We here believe that the RSS activity of HC-Pro must have greatly contributed to the viral host adaptation.

The shifts in viral diversification rates during LYSV evolution suggest that LYSVs underwent evolutionary events that changed their fitness (Fig. 7). As we demonstrated here, to understand how a pathogen adapts to its hosts, an analysis of the diversification rate of the pathogen can provide important insights into the coevolution of the pathogen and its hosts. For example, Navaud et al. (24) demonstrated that a host jump by a pathogen, which shapes its host range (i.e., host-specific adaptation), affects the shift in diversification rate for a fungal plant pathogen belonging to *Sclerotiniaceae*. Our results showed that at the lineage when the host jump occurred, the changes in diversification rate were greater in CP than in P1 and HC-Pro (Fig. 7). This may suggest that CP must have undergone great selection pressures caused by viral host adaptation to a new host driven by RSS activity of HC-Pro with P1. Our tip-dating analysis showed that the inference using P1 and HC-Pro was dated in an older evolutionary timescale than that using CP (hundreds of years difference) (Fig. 6); this supports our idea that leek LYSV’s host adaptation may have been driven by HC-Pro with P1.

To gain insight into the host factors that activate leek LYSV, we then investigated RSS activity by leek LYSV, noting that potyviral P1 and HC-Pro are viral RSSs. In our agroinfiltration experiments, the RSS activity of leek LYSV was significantly stronger than that of garlic LYSV when P1 and HC-Pro were co-expressed in leek (Fig. 9), albeit this difference was not found in onion (Fig. 8). By contrast, when *N. benthamiana* was used, garlic LYSV had stronger RSS activity than leek LYSV did (Fig. S8). These results suggest that viral RSS activity was highly host dependent. In a previous study, we showed that the P1 protein is a host determinant factor, and a deletion in the P1 N-terminal half greatly affected viral RSS activity depending on the host plant species (20).

The N-terminal half deletion coincided with the region including the repetitive motif in the P1 protein (i.e., IxFGSFETP), which was identified in this study (Fig. 5). The functional role of this unique repeat remains to be discovered, but a part of this repeat unit includes the reported potyviral motif (i.e., IxFG). Importantly, the FG motif is reported to be involved in the inhibition of stress granule formation (44). Since this repetitive motif was conserved in both leek LYSV and garlic LYSV, we were not able to show how this unique motif contributed to the host adaptation of LYSV. Nevertheless, the deletion found in this repeated motif of P1 (i.e., S-type) seems to have led to higher birth rates compared to the N-type garlic LYSV (Fig. 7), suggesting that this repetitive motif may affect viral fitness. Our previous demonstration on expanding distribution for the S-type LYSV (20) further supports this idea.

Potyvirus P1 is known as a recombination hotspot (45, 46), thus leading to gene duplication (29), which must affect the structural variation of P1 and thus its RSS activity. The P1 protein acts as a protease to cleave a polyprotein between P1 and HC-Pro, which affects viral RSS activity (29). It is worth investigating whether there is any difference in protease activity for a P1-HC-Pro fusion protein between leek and garlic isolates because, in our experiment assay, P1 and HC-Pro were expressed independently. Potyviral P1 is also reported to be involved in regulating host translation or the evasion of hormone signaling responses (47). Unraveling the effect of potyviral P1 without RSS activity on the viral host adaptation might also add some knowledge about the host adaptation of potyviruses.

A series of reports characterized the evolution of potyviruses as being mainly due to geographic isolation (23, 41, 48). On the other hand, there are very few reports that a potyvirus apparently evolved by adapting to its host (8). Our DAPC and phylogenetic analysis suggest that leek LYSV and garlic LYSV have clearly evolved independently (Fig. 3, 4, and 6). In addition, leek LYSV has not been isolated from garlic in the field; they seem not to infect garlic, supporting the hypothesis that host specificity strongly drives the adaptive evolution of LYSV. However, it is also worth noting that, at least in some areas (e.g., South America and Africa), geographic isolation contributes to the genetic segregation of LYSV to some but presumably to a lesser extent than host specificity (Fig. 3).

In summary, we demonstrated that LYSV perhaps had undergone host-specific evolution and that the viral RSS (P1 and HC-Pro) activity would play an important role in the host-adaptive evolution of the virus. LYSV could serve as an ideal model to study the process of viral host-adaptive evolution and virus-host coevolution.

## MATERIALS AND METHODS

### Plants

A garlic-like crop (later designated LG hybrid), sold as garlic, was purchased at a market in Miyazaki Prefecture, Japan. We examined four bulbs of the garlic-like crop collected from Tano town in Miyazaki prefecture, Japan. Five leek samples, cultivated by four different farmers were bought at a market in Tokachi region in Hokkaido, Japan. Four GH garlic samples, cultivated by two different farmers, were bought at a market in Saga Prefecture, Japan.

### Total RNA extraction

Total RNA was extracted from bulbs of garlic-like crop and GH garlic, and leaves of leek using TRIzol Reagent (Invitrogen, Carlsbad, CA, USA). For RNA-Seq, RNA was further purified through a column using NucleoSpin RNA (Takara, Otsu, Japan).

### RT-PCR, RNA-Seq, and quantification of viral levels

RT-PCRs were performed in either two-step or one-step reactions with 30–35 cycles. For the two-step RT-PCR, cDNA was first synthesized with AMV reverse transcriptase (Nippon Gene, Tokyo, Japan), then amplified with Takara Ex Taq (Takara). For the one-step RT-PCR, a Takara One Step RNA PCR kit (AMV) (Takara) was used. For RNA-Seq, the purified RNA was sent to Novogene (Beijing, China). The reads obtained from the RNA-Seq data were first filtered and then assembled *de novo* by Trinity v2.14.0 (49) to cover the entire LYSV genome. Viral levels were estimated by amplifying the LYSV P1 and CP sequence using RT-PCR with 40 cycles with the host actin gene as an internal control. For nested PCR, 1 µL of the first-round RT-PCR reaction mixture was added to the second-round PCR preparation. The primer sequences used are in Table S3.

### Nucleotide sequencing and assessment of genetic structure

The RT-PCR products were cloned into the pGEM T-easy vector (Promega), then the nucleotide sequences of the P1, HC-Pro, and CP genes were determined. Primers were designed based on the *de novo* assembled genomic sequences. A BLASTn search was conducted in the standard database (nucleotide collection) to identify newly obtained LYSV sequences. We then downloaded all the publicly available nucleotide sequences of LYSV from the GenBank database and curated the data set by removing any isolates that are not associated with the year of collection, sampled host, country of collection based on the information of the annotated metadata, or the original reference of each entry. After combining them with the sequence of our newly obtained isolates, a multiple sequence alignment via the translated amino acid codons for the P1, HC-Pro, NIb, and CP data set was generated using the software MUSCLE v3.8.31 (50). The translated amino acid sequences were plotted using WebLogo (https://weblogo.berkeley.edu). As a pre-processing step for the subsequent analysis, a recombination analysis was performed for the P1, HC-Pro, NIb, and CP data sets using the software RDP5 (51), and any isolates that might potentially contain some recombination sites were discarded with the criterion of *P*-value < 10^−6^ by three or more different programs implemented in the RDP5. DAPC was performed in adegenet (52) using the data set of P1 (*n* = 179), HC-Pro (*n* = 123), and CP (*n* = 300) gene with the annotation of each isolate by two independent population traits: host plant and geographic area. The number of principal components in each analysis was examined by cross-validation. The R script for the DAPC analysis is available on GitHub at https://github.com/skawakubo/Kawakubo_et_al_LYSV-leek. The list of LYSV isolates used in the analysis and each annotated population traits is summarized in Table S2.

### Bayesian phylogenetic analysis and ancestral state reconstruction

Phylogenetic analysis was performed using the Bayesian framework implemented in BEAST v2.6.7 (53). For all runs, the general-time reversible nucleotide substitution model with gamma-distributed among-site rate variation and a proportion of invariable sites (GTR + G + I) was used, along with an uncorrelated relaxed clock model with lognormal distribution and a Bayesian skyline coalescent model. The sampling year for each LYSV isolate was used to infer the evolutionary timescale of LYSV. To evaluate the temporal structure in all of the data sets in this study, we performed a date-randomization test with 10 clustered permutations (54) using the TipDatingBeast package (55). The presence of temporal structure was assumed when the 95% CI of the rate estimate from the original data set did not overlap with the 95% CI of any of the date-randomized replicates. For the prior distribution for estimating the substitution rate, the lognormal distribution (*µ* = 7.55E-4, *s* = 0.15) was applied for ucld.mean, which reasonably covers the previously reported substitution rate of another potyvirus (23). Ancestral state reconstruction was also performed with host plants of each LYSV isolate, and the transition of host states was modeled with an asymmetric substitution model. To examine whether the number of isolates in each host plant had a strong influence on the reconstruction of the host state at the root node, we compared the estimate with 10 replicates of the data set that host plants of each isolate were randomized, as suggested by previous study (23, 56). Markov chain Monte Carlo (MCMC) sampling was performed for 100,000,000 iterations, sampling every 10,000 steps. At least three independent Markov chains were run to confirm convergence. After the first 10% of samples were discarded as burn-in states, each run was combined by LogCombiner, and MCC trees were generated by TreeAnnotator within the BEAST package. The resulting trees were visualized using ggtree (57).

### Shifts in diversification rate in phylogenetic trees

Diversification dynamics of LYSV was inferred using BAMM (30) and BAMMtools (31). The BEAST MCC trees were transformed as ultrametric due to the requirement of BAMM to analyze shifts in diversification rates in the topology of the BEAST trees. MCMC sampling was performed for 5,000,000 iterations, sampling every 5,000 steps. Prior distributions were set according to the preprocessing analysis using the setBAMMpriors function, except for that the globalSamplingFraction setting was set as 0.01. For the speciation rate in the diversification model, lambda was treated as the birth rate. The R script for the BAMM analysis is available on GitHub at https://github.com/skawakubo/Kawakubo_et_al_LYSV-leek.

### RNA silencing suppression assay

The RSS activities of P1 and HC-Pro of the LYSVs were assayed using the agroinfiltration method as previously described (20, 58, 59). The expression clones were generated from an LYSV isolate of LG hybrid (LC757493) as representative of the leek strain, and an N-type LYSV isolated from garlic (LC714765), as representative of garlic strain. DNA fragments coding for the HC-Pro and FLAG-tagged P1 proteins were cloned into the pBE2113 plasmid between XbaI and SacI restriction enzyme sites, then the plasmid vectors were used to transform *Agrobacterium*. We used onion and leek as assay plants to examine the RSS activity of the viral proteins in addition to *N. benthamian*a which is a widely used experimental plant. Intact *N. benthamiana* leaves, detached onion epidermis, or leek leaves were then infiltrated with a bacterial suspension mixed with a 1:1:1 ratio of green fluorescence protein (GFP), P1, and HC-Pro constructs. The final concentration of each inoculum bacterial culture was adjusted to OD_600_ = 1.0 for the assay using *N. benthamiana* and to 0.2 for the assay using onion and leek. GFP fluorescence was examined under UV light at 5 days post-agroinfiltration (dpa) in *N. benthamiana*, and their intensity was analyzed by the ImageJ software (https://imagej.nih.gov/ij/index.html). For onion and leek, GFP intensity was quantified using LAS AF software (Leica) at 3 dpa. For all the agroinfiltration assays, five independent biological replicates were performed to confirm the results were similar. The statistical power was evaluated at the significance threshold of *P* < 0.05 by Tukey’s multiple comparison test following one-way ANOVA. The parametric test was selected by checking normality based on the Shapiro–Wilk test. To confirm the expression of the target protein, total proteins were commonly extracted using Laemmli sample buffer that contains 2% SDS with phosphate-buffered saline with 0.1% Tween 20 (PBST), from the infiltrated plant sample, and then separated using 10% SDS-PAGE. P1-FLAG was detected by western blot analysis using an anti-FLAG monoclonal antibody (FUJIFILM Wako Pure Chemical Corporation) (20, 58, 59).

## Supplementary Material

Reviewer comments

## Data Availability

Nucleotide sequence data of leek yellow stripe virus isolates obtained in this study are available in GenBank (accessions LC757493–LC757506), and the RNA-Seq data have been deposited with links to BioProject accession PRJNA996293. BEAST xml files and other computational source codes used in this study are available on the GitHub repository.

## SUPPLEMENTAL MATERIAL

Supplemental figures spectrum.02340-23-s0001.pdf Fig. S1 to S10.

Table S1 spectrum.02340-23-s0002.xlsx Comparison of the ITS regions among the *Allium* plants used in this study

Table S2 spectrum.02340-23-s0003.xlsx LYSV isolates used in our DAPC and phlogenetic analyses.

Table S3 spectrum.02340-23-s0004.xlsx Primers.

OPEN PEER REVIEW reviewer-comments.pdf An accounting of the reviewer comments and feedback.

ASM does not own the copyrights to Supplemental Material that may be linked to, or accessed through, an article. The authors have granted ASM a non-exclusive, world-wide license to publish the Supplemental Material files. Please contact the corresponding author directly for reuse.
