# Supplementary material for: Host-specific adaptation drove the coevolution of leek yellow stripe virus and Allium plants
Source: Microbiol Spectr. 2023 Sep 14;11(5):e02340-23. doi: 10.1128/spectrum.02340-23 (PMC10581216; doi:10.1128/spectrum.02340-23)
Supplement: Supplemental figures — Fig. S1 to S10. [file spectrum.02340-23-s0001.pdf]

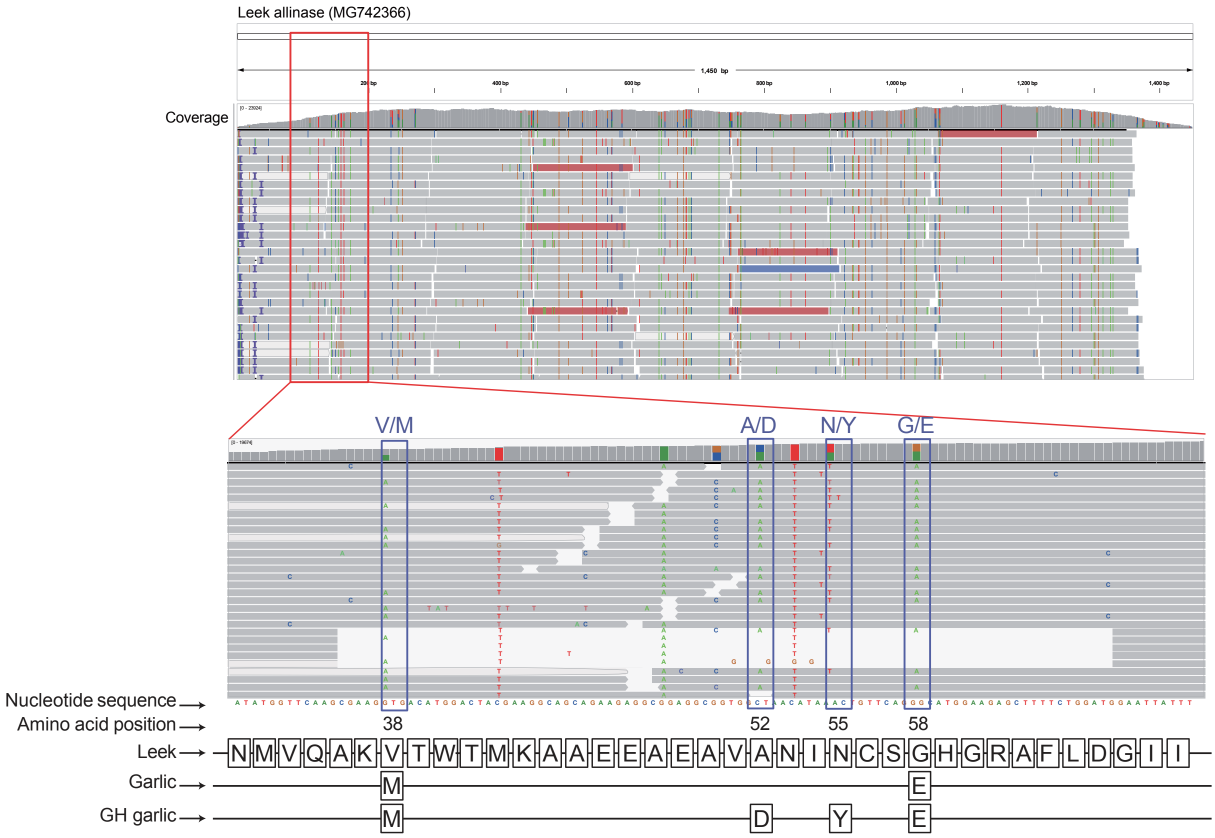

Fig. S1. Integrative Genomics Viewer (IGV) capture of the reads mapped to the alliinase gene sequences of leek (MG742366). The upper panel shows the overall mapped results, and the boxed section is magnified in the lower panel where differences in the unique amino acids among leek, garlic and GH garlic are indicated in blue squares. The amino acid sequences of the alliinase gene of leek, garlic and GH garlic are shown at the bottom. The amino acid positions are given relative to the methionine residue of the start codon. In the upper panel, reads in red indicate deletions; those in blue indicate insertions.

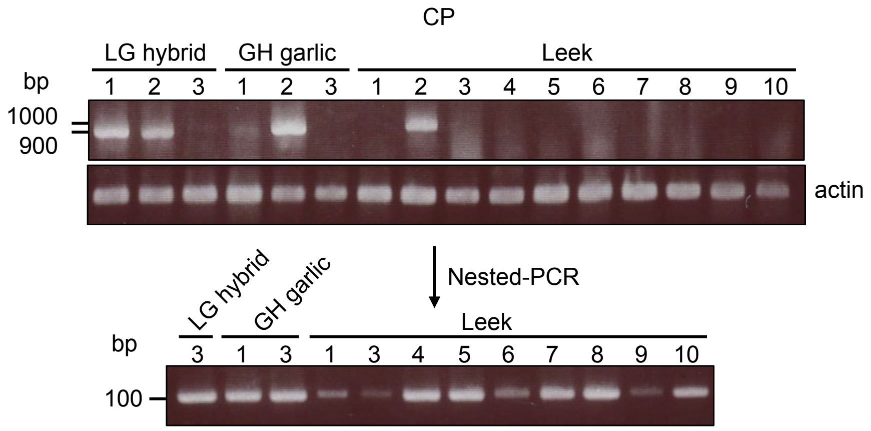

Fig. S2. RT-PCR to detect LYSV from LG hybrid, GH garlic and leek. The first-round PCR was run for 40 cycles (upper panel), then nested-PCR was run (lower panel) when no band was detected in the first-round PCR. The actin gene was used as an internal control. Lane number represents each individual plant and the number in the upper panel corresponds to those on the lower panel. PCR primers are listed in the Table S3.

A. filtered data set

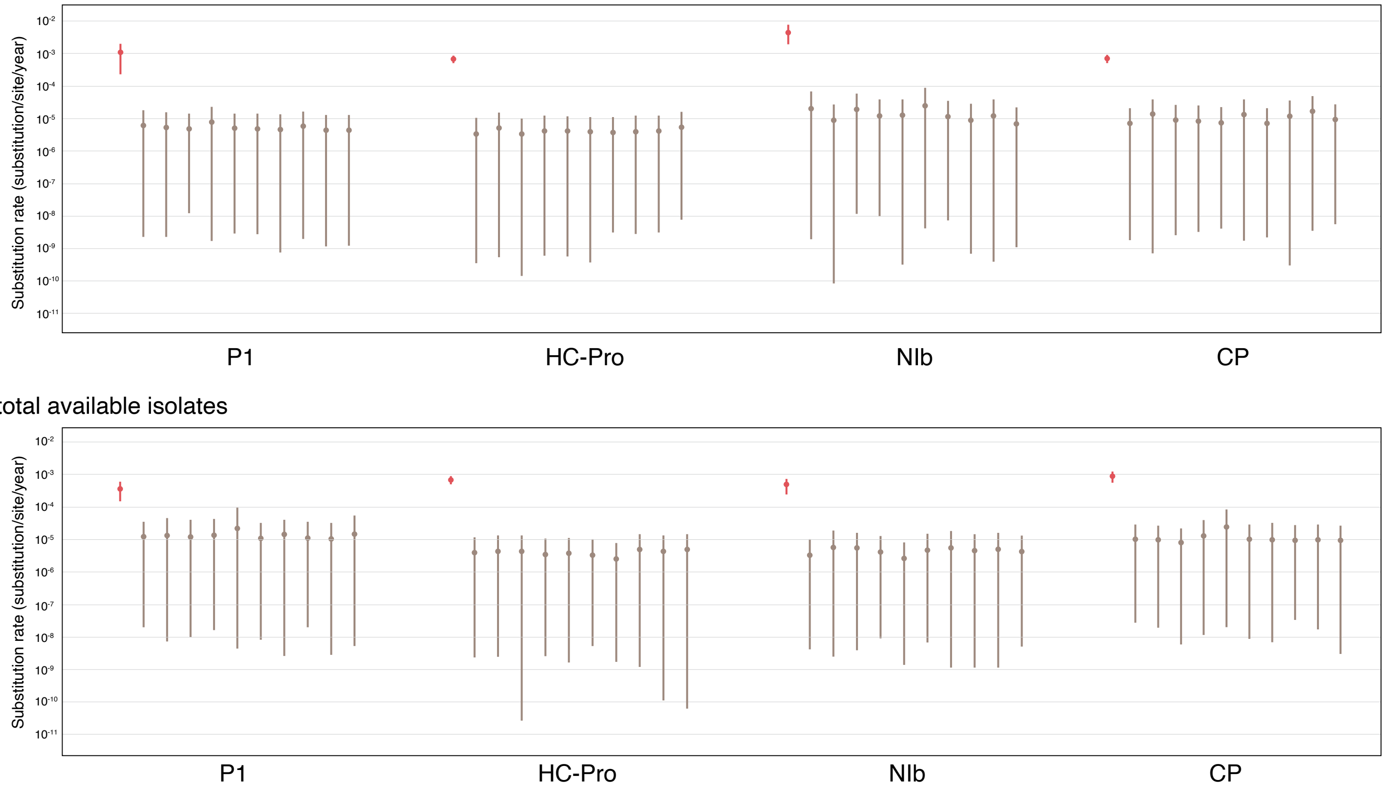

Fig. S3. Summary of the date-randomization tests for the data set used in this study. (A) Filtered data sets for four viral genes that shared the same taxon set, which were used in the Fig. 6A-C and Supplementary Fig. S9. (B) Total available isolates data sets, which were used in the Fig. 6D-F and Supplementary Fig. S10. Estimates of the nucleotides substitution rate (substitution/site/year) from the original data set and ten clustered permutations. Point symbols represent the mean rate estimate for each data set, with error bars showing the 95% credible intervals (CI). Point symbols and error bars in red color represent the estimate from the original data set, whereas those in brown color represent the estimates from the date-randomized data sets. Note that the 95% CI of the estimates from the date-randomized replicates did not overlap with the mean posterior estimate from the original data set for all the analyzed genes, indicating the presence of temporal signals.

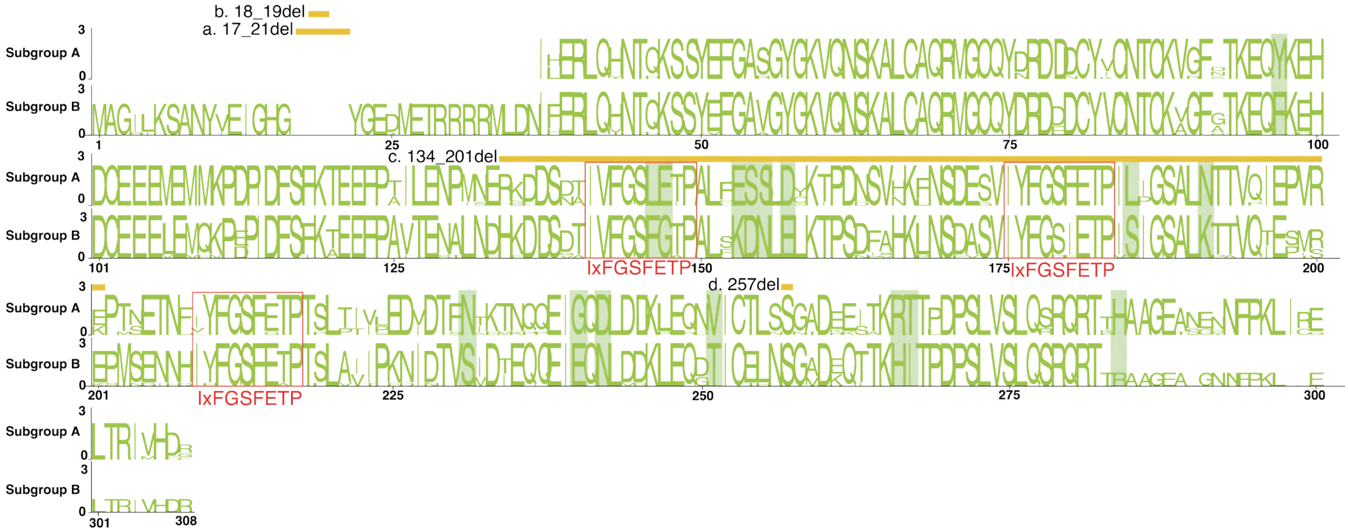

Fig. S4. Amino acid sequences of N-terminal half of P1 protein for isolates in the leek clade. The *x*-axis corresponds to the amino acid sequence position of the LYSV isolate (AB194649). The *y*-axis indicates the bit score of cross entropy at the sequence position. The yellow bar corresponds to the deletion position shown in Fig. 4. The sequence of subgroup A at the 5′ end is not shown due to the limited number of available sequences. Plots were created using WebLogo (https://weblogo.berkeley.edu).

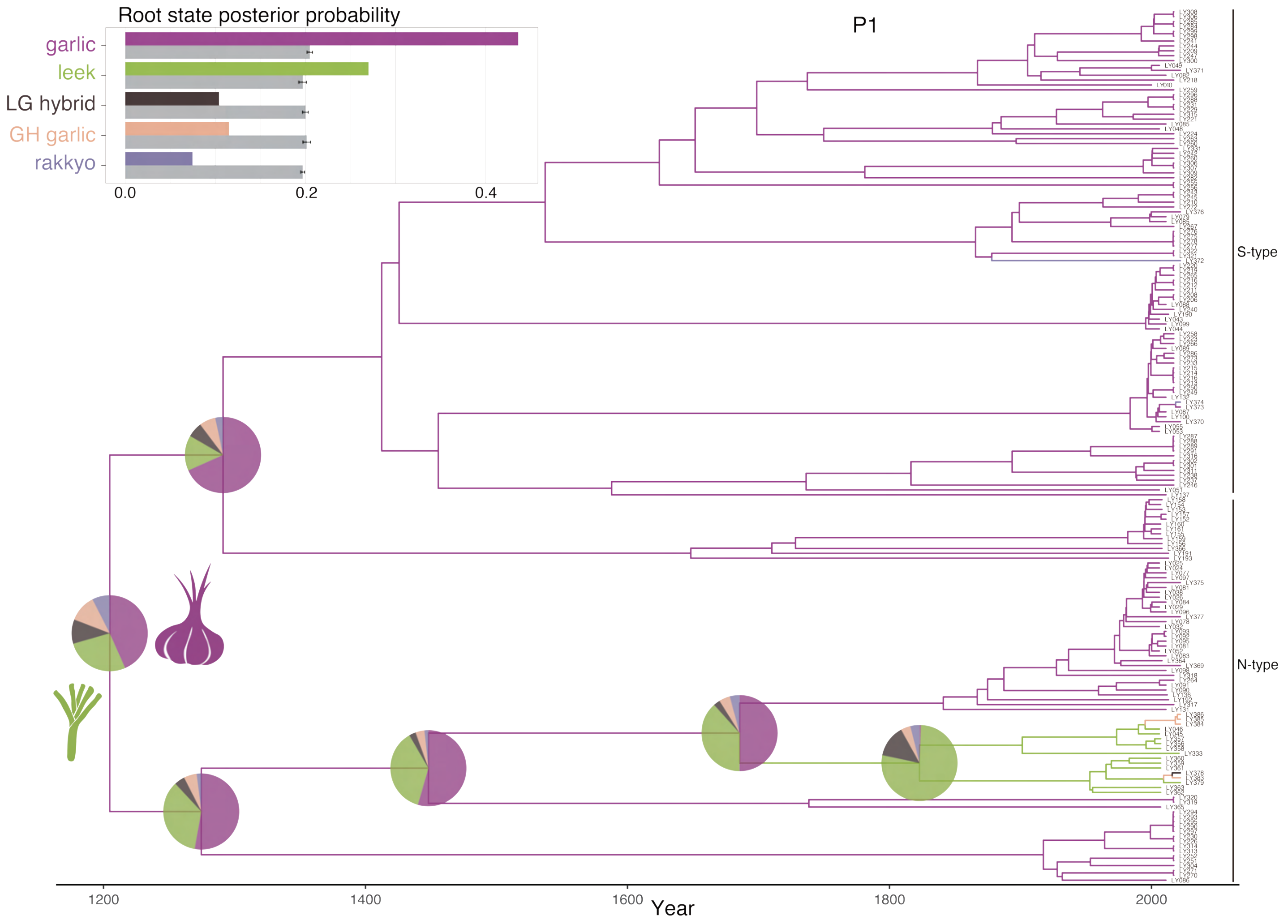

Fig. S5. Time-scaled maximum clade credibility tree for the P1 genes. This is the same tree as those in Fig. 6D, but the tip labels are annotated with the isolate index number as summarized in Supplementary Table S2. Branch colors indicate the host species in the inferred ancestral state reconstruction as defined in the key. Pie charts indicate the posterior probabilities of the host plants inferred as the ancestral state. The *x*-axis is scaled in years. The histogram (top left) shows posterior probability of the root for each host plant; the gray bars indicate the posterior probability with error bars obtained from randomized replicates of the tip state.

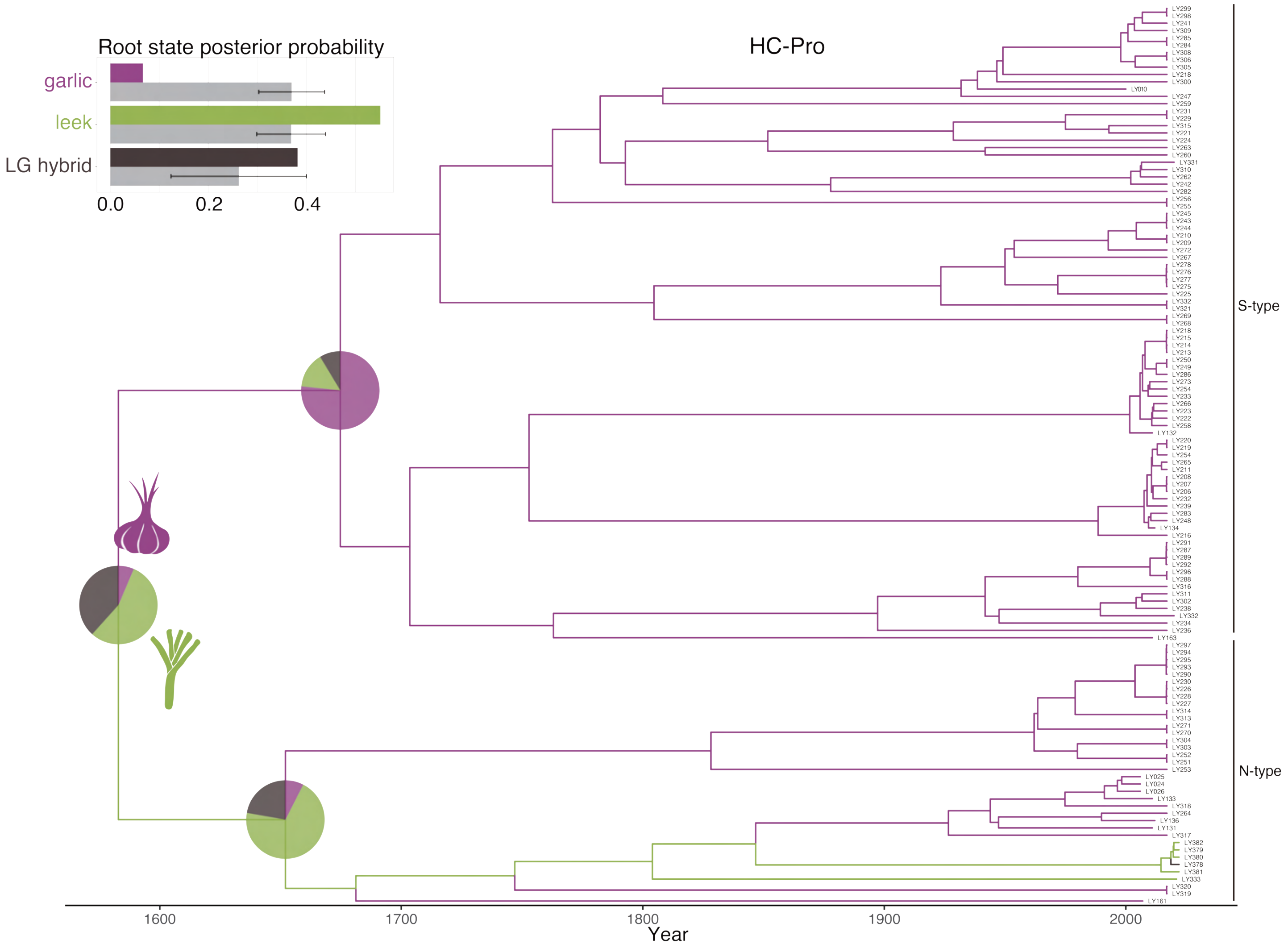

Fig. S6. Time-scaled maximum-clade credibility tree for the HC-Pro genes. This is the same tree as those in Fig. 6E, but the tip labels are annotated with the isolate index number as summarized in Supplementary Table S2. Branch colors indicate the host species in the inferred ancestral state reconstruction as defined in the key. Pie charts indicate the posterior probabilities of the host plants inferred as the ancestral state. The *x*-axis is scaled in years. The histogram (top left) shows posterior probability of the root for each host plant; the gray bars indicate the posterior probability with error bars obtained from randomized replicates of the tip state.

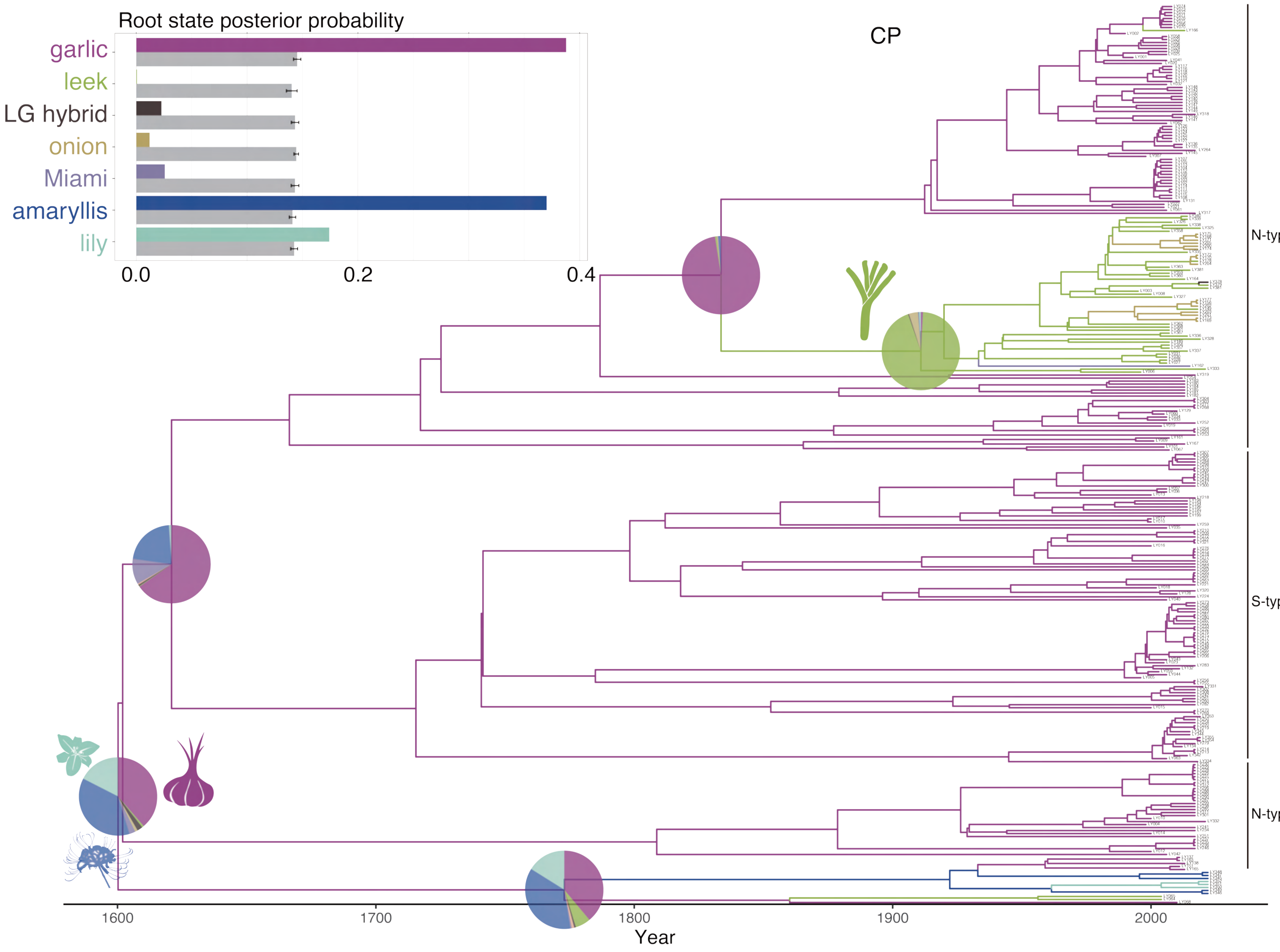

Fig. S7. Time-scaled maximum-clade credibility tree for the CP genes. This is the same tree as those in Fig. 6F, but the tip labels are annotated with the isolate index number as summarized in Supplementary Table S2. Branch colors indicate the host species in the inferred ancestral state reconstruction as defined in the key. Pie charts indicate the posterior probabilities of the host plants inferred as the ancestral state. The *x*-axis is scaled in years. The histogram (top left) shows posterior probability of the root for each host plant; the gray bars indicate the posterior probability with error bars obtained from randomized replicates of the tip state.

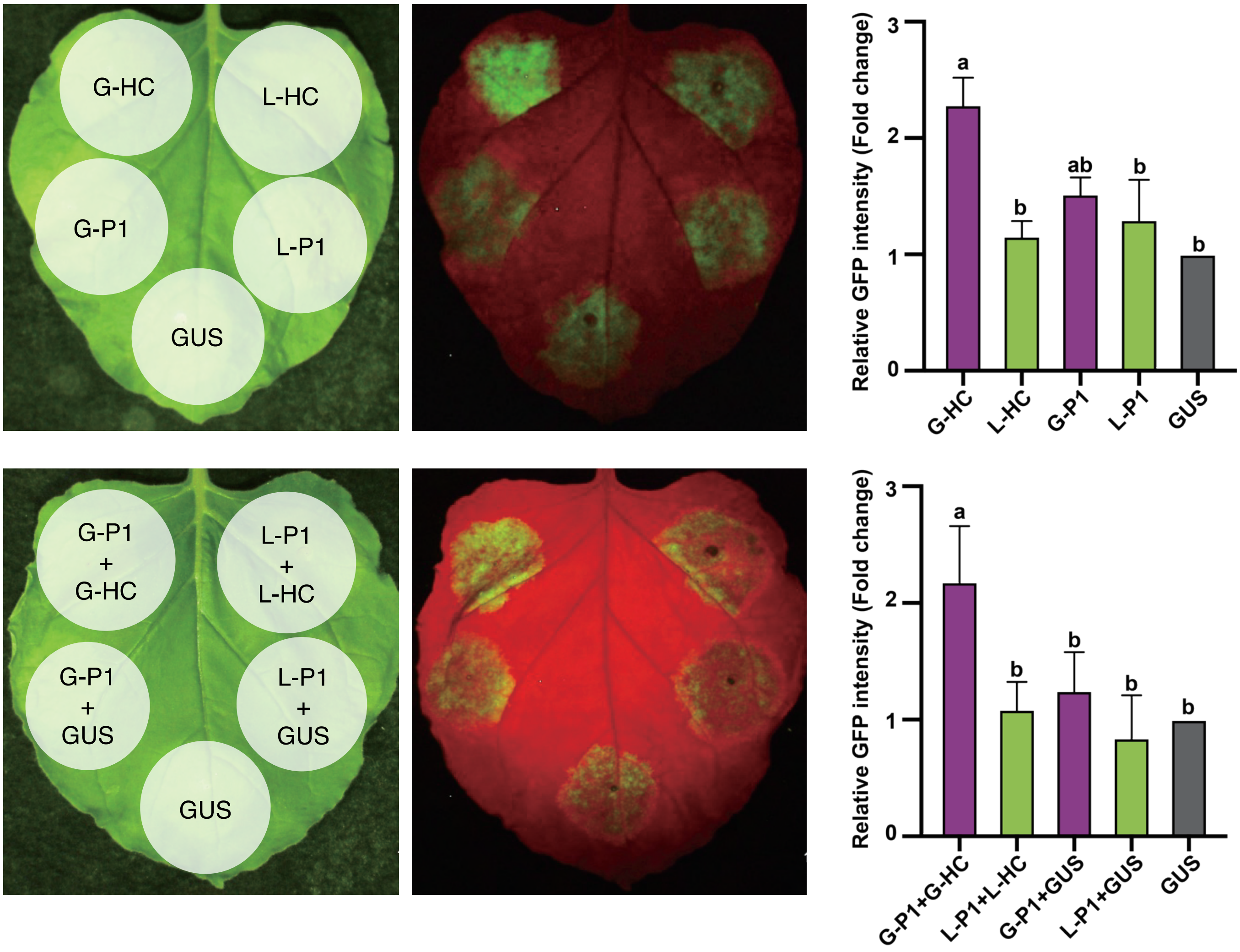

Fig. S8. RNA silencing suppressor (RSS) activity of LYSV P1 and HC-Pro at 5 days post agroinfiltration (dpa) of *Nicotiana benthamiana* leaves. The upper panels of the left pictures show the agroinfiltrated leaves that P1 and HC-Pro proteins of garlic strain (G-P1 and G-HC) and leek strain (L-P1 and L-HC) were expressed alone with the GFP gene, whereas the lower panels show that P1 and HC-Pro were co-expressed. Greater GFP fluorescence intensity indicates greater RSS activity. The *GUS* gene was used as a negative control. GFP florescence intensity was analyzed by the ImageJ software (https://imagej.nih.gov/ij/index.html). Relative mean fold-change values, when the control, GUS was set to 1.0, are shown in the barplot. Fill colors in the barplot represent the LYSV strains (purple, garlic strain; green, leek strain). One-way ANOVA test was conducted (upper plot, $P = 0.0051$; lower plot, $P = 0.0002$), followed by Tukey's multiple comparison test. Different letters above the bars indicate a significant difference between means ($P < 0.05$).

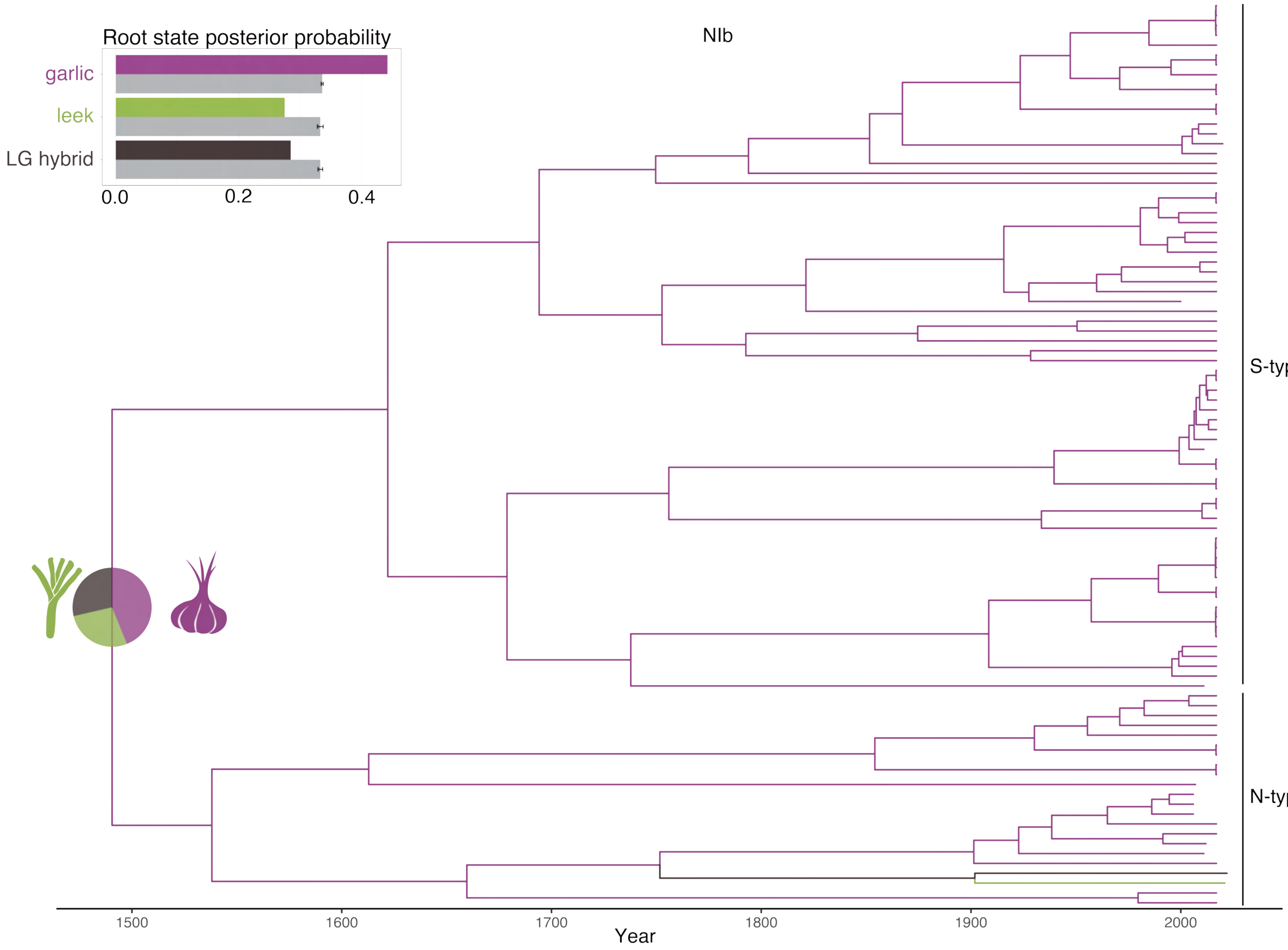

Fig. S9. Time-scaled maximum-clade credibility tree for the NIb genes based on the filtered data set (n = 92) to keep the same taxon set with the analysis shown in the Fig. 6A-C. Branch colors indicate the host species in the inferred ancestral state reconstruction as defined in the key. Pie charts indicate the posterior probabilities of the host plants inferred as the ancestral state. The x-axis is scaled in years. The histogram (top left) shows posterior probability of the root for each host plant; the gray bars indicate the posterior probability with error bars obtained from randomized replicates of the tip state.

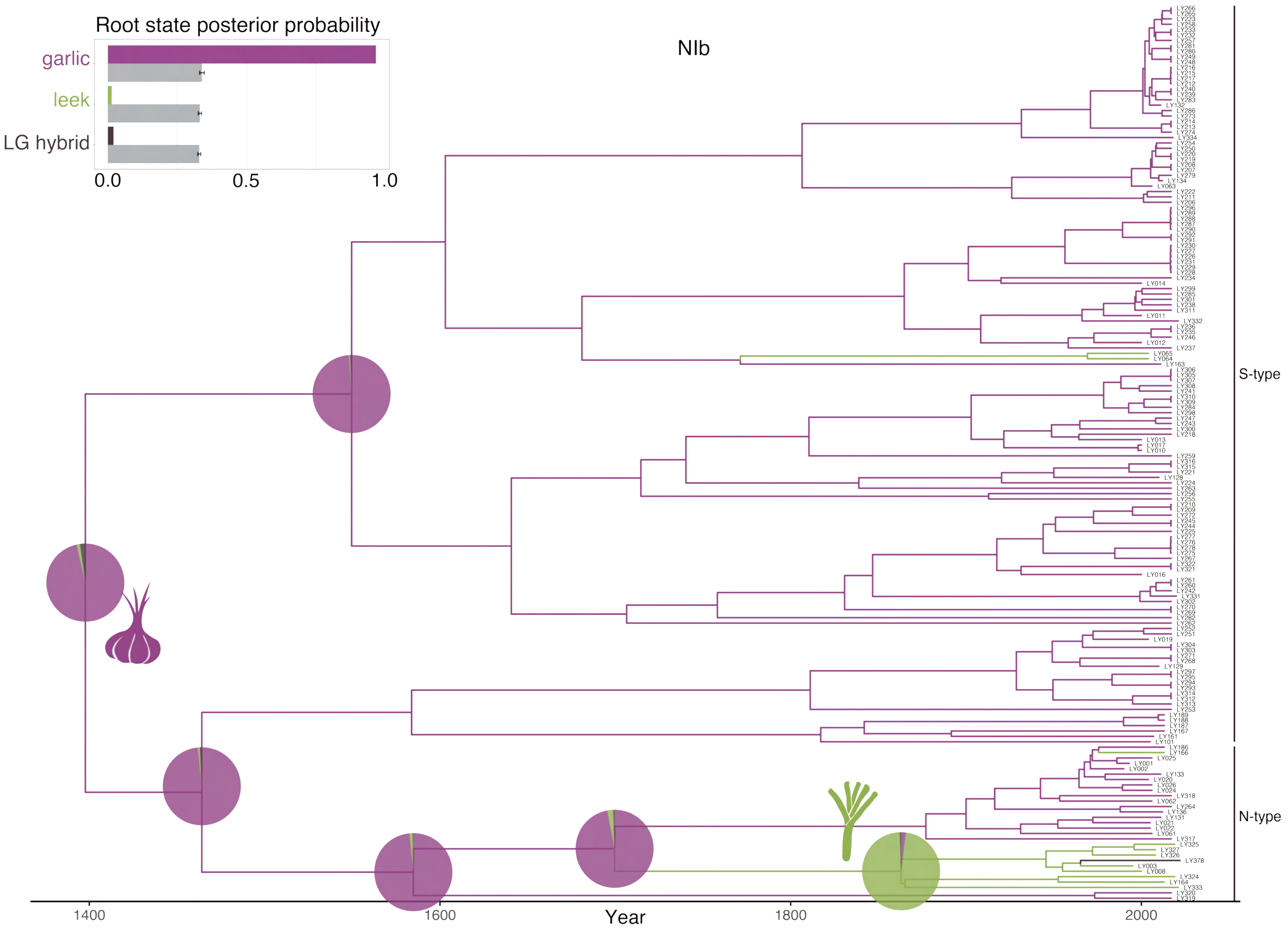

Fig. S10. Time-scaled maximum-clade credibility tree for the NIb genes. The tip labels are annotated with the isolate index number as summarized in Supplementary Table S2. Branch colors indicate the host species in the inferred ancestral state reconstruction as defined in the key. Pie charts indicate the posterior probabilities of the host plants inferred as the ancestral state. The *x*-axis is scaled in years. The histogram (top left) shows posterior probability of the root for each host plant; the gray bars indicate the posterior probability with error bars obtained from randomized replicates of the tip state.
