# Supplementary material for: Host-specific adaptation drove the coevolution of leek yellow stripe virus and Allium plants
Source: Microbiol Spectr. 2023 Sep 14;11(5):e02340-23. doi: 10.1128/spectrum.02340-23 (PMC10581216; doi:10.1128/spectrum.02340-23)
Supplement: Reviewer comments [file reviewer-comments.pdf]

AMERICAN SOCIETY FOR MICROBIOLOGY | Microbiology Spectrum

Host-specific adaptation drove the coevolution of leek yellow stripe virus and *Allium* plants

Shusuke Kawakubo, Hangil Kim, Minoru Takeshita, and Chikara Masuta

Corresponding Author(s): Shusuke Kawakubo, Hokkaido Daigaku

Review Timeline:

	Submission Date:	June 26, 2023
	Accepted:	July 11, 2023

Editor: Lindsey Burbank

Reviewer(s): The reviewers have opted to remain anonymous.

Transaction Report:

(Note: With the exception of the correction of typographical or spelling errors that could be a source of ambiguity, letters and reports are not edited. The original formatting of letters and referee reports may not be reflected in this compilation.)

DOI: https://doi.org/10.1128/spectrum.02340-23

July 11, 2023

Mr. Shusuke Kawakubo
Hokkaido Daigaku
Research Faculty of Agriculture
Kita-9, Nishi-9
Kita Ward
Sapporo, Hokkaido 060-8589
Japan

Re: Spectrum02340-23 (Host-specific adaptation drove the coevolution of leek yellow stripe virus and *Allium* plants)

Dear Mr. Shusuke Kawakubo:

Your manuscript has been accepted, and I am forwarding it to the ASM Journals Department for publication. You will be notified when your proofs are ready to be viewed.

The ASM Journals program strives for constant improvement in our submission and publication process. Please tell us how we can improve your experience by taking this quick Author Survey.

Publication Fees: We have partnered with Copyright Clearance Center to collect author charges. You will soon receive a message from no-reply@copyright.com with further instructions. For questions related to paying charges through RightsLink, please contact Copyright Clearance Center by email at ASM_Support@copyright.com or toll free at +1.877.622.5543. Hours of operation: 24 hours per day, 7 days per week. Copyright Clearance Center makes every attempt to respond to all emails within 24 hours. For a complete list of **Publication Fees**, including supplemental material costs, please visit ourwebsite.

ASM policy requires that data be available to the public upon online posting of the article, so please verify all links to sequence records, if present, and make sure that each number retrieves the full record of the data. If a new accession number is not linked or a link is broken, provide production staff with the correct URL for the record. If the accession numbers for new data are not publicly accessible before the expected online posting of the article, publication of your article may be delayed; please contact the ASM production staff immediately with the expected release date.

Corresponding authors may join or renew ASM membership to obtain discounts on publication fees. Need to upgrade your membership level? Please contact Customer Service at Service@asmusa.org.

Thank you for submitting your paper to Spectrum.

Sincerely,

Lindsey Burbank
Editor, Microbiology Spectrum

Journals Department
American Society for Microbiology
1752 N St., NW
Washington, DC 20036
E-mail: spectrum@asmusa.org
